# Diagnostic and Predictive CSF Biomarkers in Idiopathic Normal Pressure Hydrocephalus (iNPH): A Systematic Review

**DOI:** 10.3390/ijms27208986

**Published:** 2026-10-10

**Authors:** Emanuela Procopio, Arianna Accattatis, Carmelino Angelo Stroscio, Giusy Guzzi, Angelo Lavano, Domenico La Torre, Attilio Della Torre

**Affiliations:** 1 Division of Neurosurgery, Department of Biomedical, Dental and Morphological and Functional Imaging, University of Messina, Via Consolare Valeria, 98125 Messina, Italy; emanuela.procopio@studenti.unime.it (E.P.); arianna.accattatis@studenti.unime.it (A.A.); 2 Division of Neurosurgery, Department of Medical and Surgical Sciences, University Magna Graecia of Catanzaro, 88100 Catanzaro, Italy; carmelinoangelo.stroscio@aourenatodulbecco.it (C.A.S.); giusy.guzzi@aourenatodulbecco.it (G.G.); lavano@unicz.it (A.L.); 3 Neurosurgical Unit, Department of Pharmacy, Health and Nutrition Sciences, University of Calabria, 87036 Rende, Italy; domenico.latorre@unical.it

**Keywords:** idiopathic Normal Pressure Hydrocephalus (iNPH), CSF biomarkers in iNPH, VP shunt outcome prediction, NPH differential diagnosis, glymphatic clearance in hydrocephalus, amyloid and Tau dynamics in CSF, neuroinflammation in iNPH, CSF lipidomics and proteomics

## Abstract

Idiopathic normal pressure hydrocephalus (iNPH) is a progressive disorder clinically characterized by Hakim’s triad: gait disturbance, cognitive impairment, and urinary incontinence. iNPH treatment consists of neurosurgical implantation of a ventriculoperitoneal CSF diversion, whose outcome depends on an early and correct diagnosis. Currently, there is no specific diagnostic test to perform preoperatively. Still, the combination of neurological examination, gait analysis, neurocognitive evaluation, and the tap test is useful to suggest when surgery is indicated. Our systematic review aims to identify diagnostic and outcome predictor CSF biomarkers (BM) in iNPH. The present systematic review was conducted following PRISMA 2020 guidelines. PubMed, Scopus, and the Cochrane Library databases were first consulted in April 2025 and updated in May 2026. Studies were selected using the terms “idiopathic normal pressure hydrocephalus” and “CSF biomarkers” under Medical Subject Headings (MeSH) as inclusion criteria. The eligibility of a record was defined according to the study title and abstract. The QUADAS-2, QUIPS, ROBINS-I V2, and RobVis tools were used to assess the risk of bias. Ninety-nine records were included in this systematic review. Amyloid proteins (Aβ40-42), followed by tau (τT, τP−181) and neuroinflammatory proteins were the most frequently CSF BM analyzed in patients with iNPH. ELISA was the most used method to measure BM in CSF samples. Forty-seven records investigated the potential diagnostic role of CSF BM; comparisons were frequently made among iNPH, Alzheimer’s disease (AD), and healthy control CSF samples. Regarding outcome prediction, 28 records examined CSF BM, with heterogeneous comparisons such as lumbar versus ventricular CSF or shunted versus non-shunted iNPH CSF samples, including healthy controls and iNPH. Seven records proposed both the diagnostic and predictive role of some CSF biomarkers, especially for tau proteins and Aβ42. On the other hand, 17 records concluded that neither a diagnostic nor a predictive role could be proposed for the CSF BM they analyzed. However, these records increased knowledge about an iNPH CSF pattern. Collecting and analyzing CSF samples from iNPH patients can be advantageous in differential diagnosis with other similar neurodegenerative disorders, especially AD, and therefore a predictor of neurosurgical outcomes. Combined biomarker panels should be integrated in standardized protocols, measuring: Aβ42, Aβ42/Aβ40, p-tau, total tau, ADAβ42/p-tau, L-PGDS, NFL and NRGN.

## 1. Introduction

Idiopathic normal pressure hydrocephalus (iNPH) is a progressive condition observed typically in the elderly population, clinically presenting with one or more elements of the Hakim–Adams’ triad: gait disturbance, urinary incontinence, and cognitive decline, with an estimated prevalence of up to 5.9% in individuals aged 80 years and older. Clinical diagnosis remains particularly challenging due to substantial symptom overlap with mimicking neurodegenerative conditions, such as Alzheimer’s disease (AD) and Parkinsonian syndromes. Although classical management relies on ventriculoperitoneal (VP) shunting, current surgical options have expanded to include lumboperitoneal (LP) shunting, ventriculo-atrial (VA) shunting, endoscopic third ventriculostomy (ETV), and emerging micro-invasive technologies such as the eShunt system. Identifying reliable cerebrospinal fluid (CSF) biomarkers is therefore critical to optimizing differential diagnosis, predicting surgical responsiveness, and tracking post-treatment outcomes. Based on the algorithm provided by the third edition of the Japanese Society of Normal Pressure Hydrocephalus Guidelines, we can preoperatively classify patients as “possible”, “probable”, or “improbable” iNPH. Only patients who respond to shunt surgery, clinically improving postoperatively, can be clearly diagnosed as “definite iNPH” (Nakajima, 2021) [1]. Correct patient selection before ventriculoperitoneal (VP) shunting is crucial to obtain a favorable outcome. This suggests a real diagnostic struggle, because there is no specific diagnostic exam to perform. Instead, there are different tests to put together like the pieces of a puzzle. Preoperative tests include neurological examination, gait analysis, neurocognitive evaluation, and tap tests. This last one could be useful not only to evaluate clinical improvement after cerebrospinal fluid (CSF) removal but also to qualitatively evaluate CSF dosing and possible diagnostic and/or predictive biomarkers (BM). Our systematic review aims to analyze current literature to identify possible diagnostic as well as outcome predictive CSF BM for iNPH.

## 2. Materials and Methods

### 2.1. Evidence Acquisition

The present systematic review was conducted following the PRISMA 2020 guidelines (Page M J, 2021) [2] integrated with the Synthesis Without Meta-analysis (SWiM) guidelines (Campbell M, 2020) [3]. PubMed, Scopus, and the Cochrane Library databases were first consulted in April 2025. The data were uploaded at the end of May 2026. Studies were selected using “idiopathic normal pressure hydrocephalus” and “CSF biomarkers” as terms in the Medical Subject Headings (MeSH) as inclusion criteria. When MeSH terms were not available in Advanced Search, the above combination terms were searched within Article Title, Abstract, and Keywords. To ensure a comprehensive retrieval of the relevant literature, the search strategy incorporated terms covering all established and emerging surgical interventions for iNPH, including ventriculoperitoneal (VP), lumboperitoneal (LP), ventriculoatrial (VA) shunting, endoscopic third ventriculostomy (ETV), and novel endovascular CSF diversion techniques (eShunt). Studies evaluating CSF biomarkers in patients undergoing any of these surgical modalities were eligible for inclusion. The search strategy, based on the choice of searching for “iNPH” and “CSF biomarkers”, gave us the opportunity to include a variety of papers examining CSF biomarkers, whether collected by tap test, ELD, or intraoperatively, and proposing a diagnostic role as well as a predictive, both, or neither of them. Article language was considered a limit and therefore an exclusion criterion. We included only studies written in English. Studies were then grouped according to study type. The article type filter was used to subgroup results into meta-analyses, systematic reviews, reviews, clinical trials, and other study types. The results were screened by two authors (E.P. and A.D.T.). Duplicate results across databases were removed, considering each paper only once. To avoid counting the same evidence more than once, when it comes to considering primary studies and reviews, we reported primary studies’ data results in tables, while the reviews in which those primary studies may be included are just cited in the manuscript. The eligibility of a record was defined according to the study’s title and abstract. Eligible records underwent full-text analysis when available. We recognize that excluding papers because of full text unavailability could lead to selection bias, and therefore it can be considered as a limit. Data were collected from each report by a single author (E.P.) and reviewed independently by four authors (A.D.T., C.A.S., G.G., and A.A.). Final revisions were made by two authors (A.L., D.L.T.) Data collected included the type of study, aim of study, population variables, outcomes, and methodology used. Study outcomes considered were:

- iNPH CSF biomarkers qualitative and quantitative measurable
- iNPH diagnostic CSF biomarkers that could be used as a differential diagnosis tool from other neurodegenerative conditions, especially Alzheimer’s Disease (AD)
- iNPH CSF biomarkers as a predictor of shunt surgery outcome.

Studies were grouped for synthesis according to the outcome in the following categories: Papers proposing diagnostic BM, predictive BM, both, or neither of them. The standardized metric considered was *p*-value because of the heterogeneity of parameters, in which it was the only comparable parameter. The synthesis method consisted of reporting, explaining, and comparing data, summarizing major evidences per group, and combining *p*-values. The results of papers assessed to have a low risk of bias were prioritized. Heterogeneity was investigated by informal methods, and the results are reported in tables due to the effects of modifiers such as methodological characteristics, subpopulations, and contextual factors in the studies. The certainty of the evidence was assessed considering the massive number of studies and participants. The risk of bias was also assessed. Collected data are presented in the text, reported and compared. Complete open access data are visually displayed in Table 1’s quantitative comparison of “normal pressure hydrocephalus” and “CSF biomarkers”. Combination searches versus individual searches of “idiopathic normal pressure hydrocephalus” and “CSF biomarkers” were also made to underline the importance of differentiating iNPH from secondary NPH.

### 2.2. Risk of Bias Assessment

The included studies underwent a thorough risk of bias assessment. Because of the different outcomes considered in this review, the records were divided into descriptive studies and studies proposing iNPH CSF biomarkers as diagnostic or outcome predictors. Because of that, different bias assessment tools were used as follows:

- The QUADAS-2 tool was used for bias assessment in diagnostic test accuracy studies.
- The QUIPS tool was used for bias assessment in prognostic accuracy studies.
- The ROBINS-I tool was used for bias assessment in descriptive studies.

Then, the RobVis tool (version 0.3.0) was used to synthesize the risk of bias assessment visually (McGuinness, 2020) [4].

## 3. Results

### 3.1. Evidence Synthesis Results

**Figure 1:**
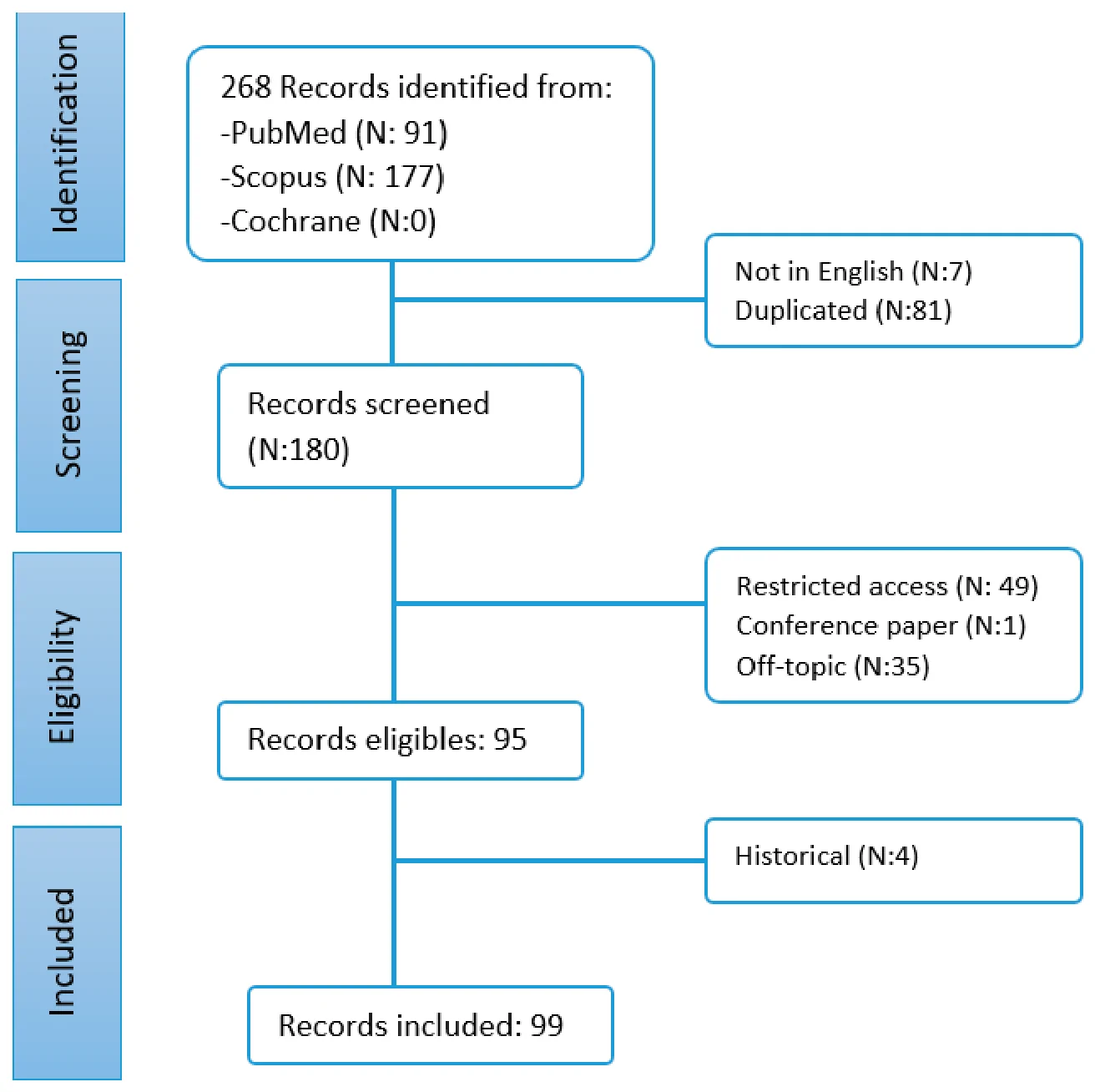
Records selection process.

The PubMed database results from a MeSH term search for “idiopathic normal pressure hydrocephalus” and “CSF biomarkers” listed 91 records published between 2004 and 2026, 89 of which were written in English. After filtering by article type, the list included four comparative studies, one multicenter study, two observational studies, 1 clinical trial, and 11 reviews (three of which were classified as systematic reviews, one of which was also classified as a meta-analysis). The remaining record types were unclassified. A Scopus combined search of “idiopathic normal pressure hydrocephalus” and “CSF biomarkers” by article, abstract, and keywords showed a total of 177 records from 2006 to 2026, 172 of which were written in English. Analyzing results by document type, 148 records were classified as articles, while 21 records were classified as reviews. The remaining three were classified as letters, book chapters, and conference papers. Lastly, in the Cochrane Library, the Title, Abstract, and Keyword search for “idiopathic normal pressure hydrocephalus” and “CSF biomarkers” resulted in four English-language trials. In contrast, the MeSH search for “idiopathic normal pressure hydrocephalus” was unavailable, producing no results. We identified a total of 268 records. Seven records were excluded because they were not written in English. We found 81 duplicated studies across different databases, each of which was considered once. Then, 180 records were screened. Eligibility was defined according to papers’ full-text availability and whether the title and abstract correlated with the topic of our systematic review. One hundred and thirty-one records were open access and free full-text available; 36 records were removed. Ninety-five records were considered eligible. Another four records were included for historical importance, although they were not available in open access. At the end of the process, a total of ninety-nine records were included. (Figure 1) We also quantitatively compared records resulting from searching the combination “normal pressure hydrocephalus” and “CSF biomarkers” on these databases with records resulting from specifically searching “idiopathic normal pressure hydrocephalus” and “CSF biomarkers”. We found that limiting our search to iNPH, rather than considering even secondary NPH, reduced the records available by 106, 104 of which were written in English. In fact, not limiting the search to iNPH, we found on PubMed 121 records (118 of which were in English) and on Scopus 245 records (239 in English). On the Cochrane Library, the Title, Abstract, and Keyword search of “normal pressure hydrocephalus” and “CSF biomarkers” resulted in nine English-language trials, and “normal pressure hydrocephalus” as a MeSH descriptor with “cerebrospinal fluid” qualifier led to four results from 2013 to 2021 made up of one Cochrane review and three trials, all written in English. This comparison underlines the importance of differentiating iNPH from secondary NPH in the literature (Table 1).

**Figure 2:**
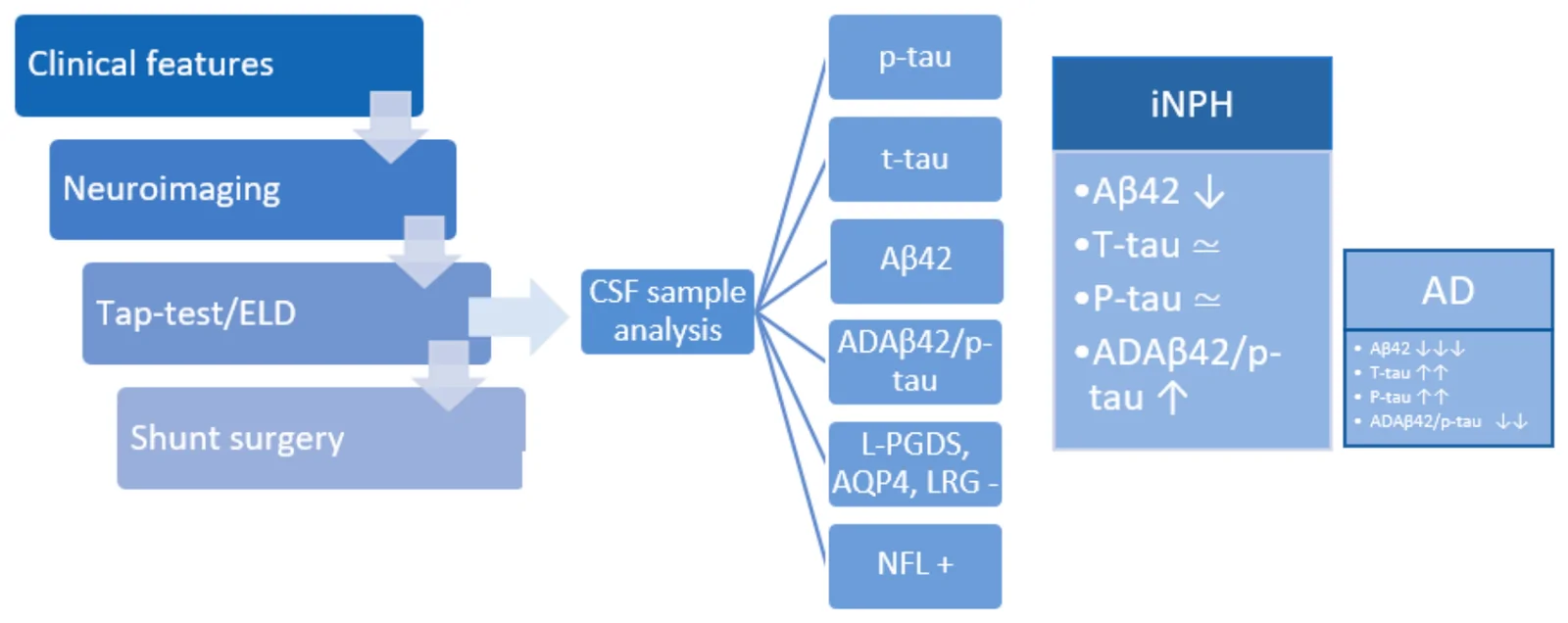
Integrated algorithm and biomarker panel of iNPH CSF BM and its main differences with AD.

Finally, among the 99 records included in this systematic review, amyloid proteins (Aβ40-42) followed by tau (τT, τP−181) and neuroinflammatory proteins were the most frequently CSF BM analyzed in patients with iNPH. An integrated algorithm and biomarker panel of useful CSF BM for iNPH is reported in Figure 2. Biomarker candidates retrieved across the reviewed literature exhibit structural differences based on their physical state in the CSF matrix. The vast majority of evaluated markers (e.g., core amyloid peptides, total and phosphorylated tau, neurofilament light chain) represent soluble/free molecules quantified via standard enzyme-linked immunosorbent assay (ELISA) or multiplex immunoassay platforms. Conversely, a distinct subset of emerging studies has focused on extracellular vesicle (EV)-bound proteins, specifically microvesicles and exosomes derived from neural and glial cells. EV-encapsulated proteins provide isolated insights into cell-specific pathological strain and metabolic dysfunction, demonstrating distinct diagnostic performance compared to their free-floating soluble counterparts in CSF. It should be noted that to date, primary literature specifically investigating EV-associated biomarkers in iNPH remains scarce, and this distinction is drawn from a still-limited body of emerging studies rather than an established body of iNPH-specific evidence.

Total and phosphorylated tau levels are generally normal in iNPH. Aβ42 is decreased with a low ADAβ42/p-tau ratio. Levels of AQP4, LRG, and L-PGDS tend to be low preoperatively, while high preoperative NFL are reported. ELISA was the most used method to measure BM in CSF samples. Forty-seven records investigated the potential diagnostic role of CSF BM, frequently comparing iNPH, Alzheimer’s disease (AD), and healthy control CSF samples. Twenty-eight records examined possible predictive CSF BM, with heterogeneous comparisons such as lumbar versus ventricular CSF or shunted versus non-shunted iNPH CSF samples, including healthy controls and iNPH. Seven records proposed both the diagnostic and predictive role of some CSF biomarkers, such as tau proteins and Aβ42. On the other hand, 17 records reported neither a diagnostic nor a predictive role could be proposed for the CSF BM they analyzed. However, these records increased knowledge about an iNPH CSF pattern. We report the CSF biomarkers analyzed in the literature considered in this review in Table S1—Supplementary File.

### 3.2. Risk of Bias Assessment Results

**Figure 3:**
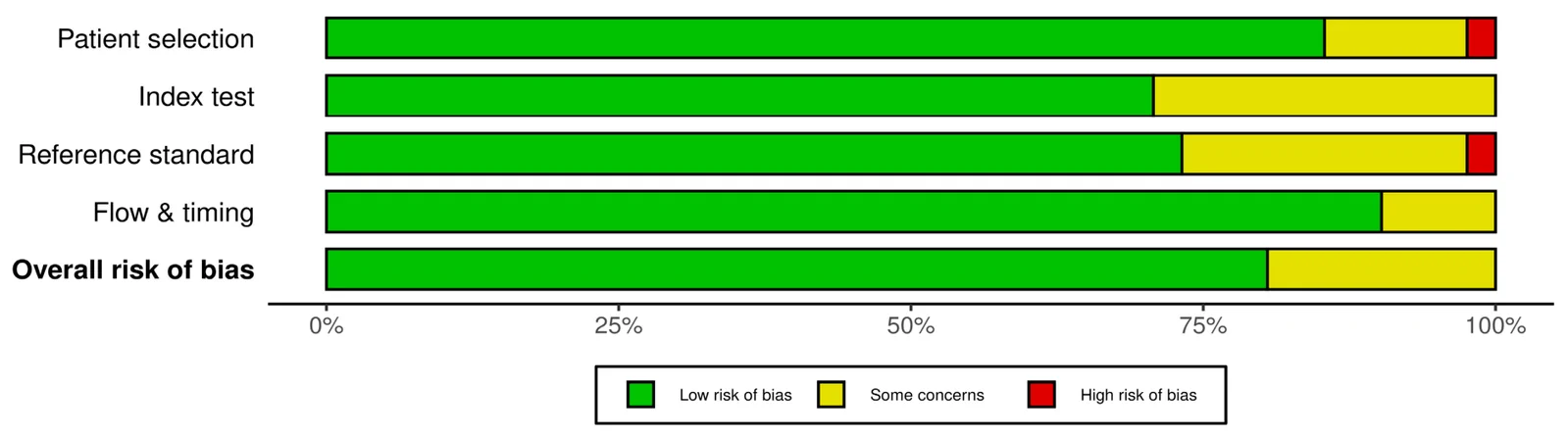
QUADAS-2 summary plot.

**Figure 4:**
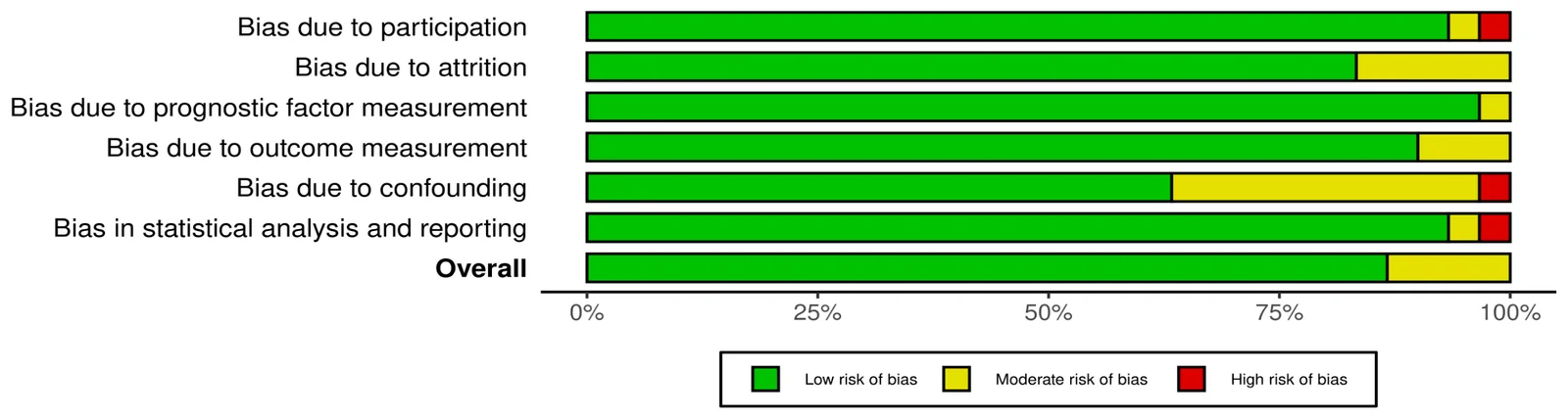
QUIPS summary plot.

**Figure 5:**
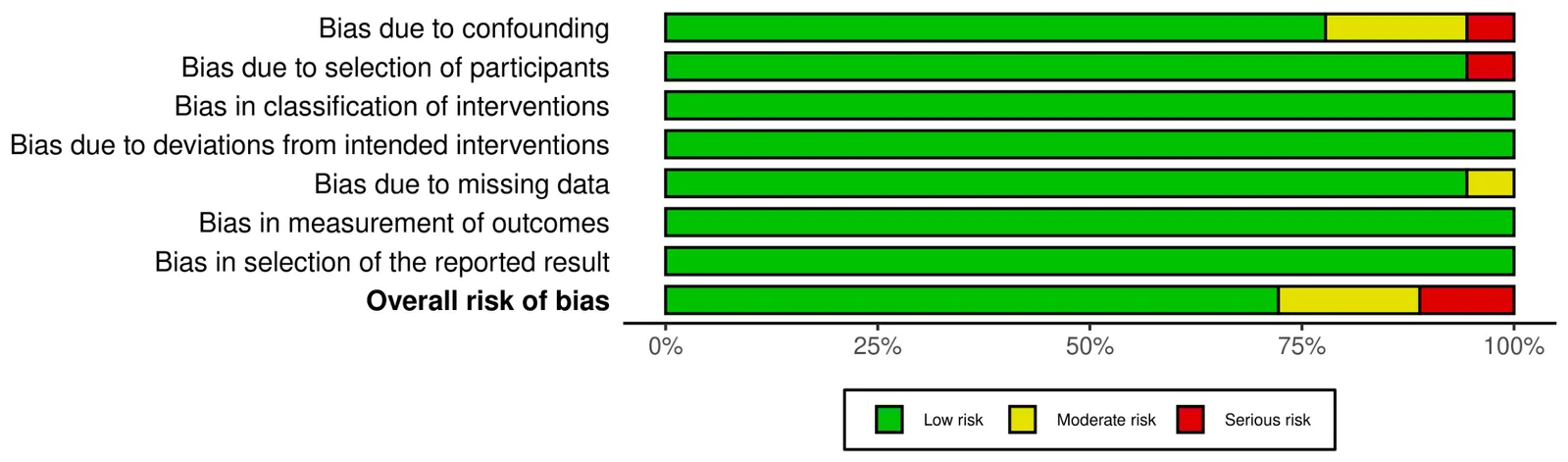
ROBINS-I summary plot.

The QUADAS-2, QUIPS, ROBINS-I tools were used to assess the risk of bias. The quality assessment is visually shown with summary bar plots of the distribution of risk of bias judgements within each bias domain. We compiled three summary bar plots, one for each risk of bias assessment tool used, generated by the RobVis tool. The QUADAS-2 (Figure 3) and QUIPS (Figure 4) results showed an overall low risk of bias, with over 75% of the papers falling into this category, and less than 25% of some concern and moderate risk of bias. However, the patient selection domain in QUADAS-2 showed higher risk of bias, highlighting once more the difficulty of iNPH patient identification. Particularly relevant is QUIPS domain results interpretation: Over 80% low attrition risk of bias coexisted with about 65% low and a 5–10% high risk of confounding bias, suggesting how confounding factors (e.g., comorbid AD) impact the validity of the shunt response predictions. The ROBINS-I (Figure 5) results also show an overall low risk of bias but with a lower percentage, around 70%, with the remaining 30% representing moderate and serious risk of bias. This aspect is not concerning considering that this tool was used for descriptive studies not proposing CSF BM as either a diagnostic or prognostic tool. Finally, in consideration of the overall risk of bias assessment results, we can conclude our systematic review conclusions are based primarily on studies demonstrating a low risk of bias.

## 4. Discussion

The clinical translation of CSF biomarkers in iNPH requires a nuanced understanding of dynamic clearance pathways and structural tissue strain. Disruption of the glymphatic system—mediated by perivascular pathways and astrocytic aquaporin-4 (AQP4) polarization—represents a primary mechanism in iNPH pathogenesis, leading to transependymal CSF stasis and reduced parenchymal flushing [5]. This mechanism explains why reduced CSF Aβ42 in iNPH reflects altered clearance rather than heavy cortical plaque deposition, maintaining a relatively preserved Aβ42/Aβ40 ratio and normal tau levels in pure iNPH compared to AD co-pathology. Furthermore, baseline elevations in neurofilament light chain (NfL) and neuroinflammatory cytokines (MCP-1, LRG) reflect ongoing white matter compression and ependymal injury. Assessing these markers within a multi-analyte matrix provides essential diagnostic precision and prognostic guidance for surgical selection. Among the literature analyzed, the most frequently CSF biomarkers examined are amyloid-β oligomers and tau proteins. The former are soluble free protein fragments floating in CSF, strongly neurotoxic and associated with cognitive impairment. Tau proteins are usually intraneuronal proteins, and they are released in CSF after neuronal and axonal damage. They can be found in CSF either as free monomers or bound to microvescicles, reflecting the neurodegenerative process. In particular, total Tau values are neuronal/axonal damage markers, and phosphorylated tau is a neurofibrillary degeneration marker, more specific for AD. Because of CSF accumulation, iNPH lacks toxic metabolite wash-out, such as Aβ42. Regarding this aspect, one of the most promising etiological theories underlying iNPH is an inefficient glymphatic system that could also explain altered Aβ clearance pathways. The glymphatic system is the brain’s drainage and waste clearance pathway, managing the circulation of CSF throughout it. This pathway works mainly during deep sleep, when synaptic downscaling or pruning also happen. Poor sleep would reflect on poor glymphatic clearance playing a role in neurodegenerative disease, an especially relevant aspect in iNPH, in which transient spikes of increased intracranial pressure are registered during REM phases. Interesting findings include neuroinflammatory pathways, especially the neurofilament light protein (NFL), a cytoskeletal protein fragment acting as an axonal injury marker, because of its release in CSF as free soluble protein fragment (like tau proteins) after axonal injury. Also remarkable is the leucine-rich α2-glycoprotein (LRG) role, which is a soluble free-secreted extracellular glycoprotein whose release by astrocytes is induced by inflammation. It has been found to increase in the elderly, in neuroinflammation, and in some neurodegenerative disorders. Another biomarker investigated is the lipocalin-type prostaglandin D synthase (L-PGDS), a CSF soluble free-secreted extracellular protein produced by arachnoid cells acting like chaperones preventing neurotoxic aggregations of amyloid beta proteins. L-PGDS is found at low levels in iNPH patients. A combination of all of these biological mechanisms may underlie iNPH pathophysiology, but the effects are as yet unclear, therefore dictating the “idiopathic” condition. ELISA was the most used method to measure BM in CSF samples. Other types of techniques were less frequently used, mostly depending on the biomarker considered. Immunoassays other than ELISA that were applied were ECLIA and CLEIA. The advanced multiplex methods used were multiplex immunoassay and PEA. Among the proteomics and mass spectrometry methods implied figured MALDI and LC-MS. Inter-assay variability should be considered. It could depend on many factors, such as analyte, calibration and standardization, environment, operators, instruments, reagent batches, different dynamic ranges, and cross-reactivity noise.

### 4.1. iNPH Diagnostic CSF Biomarkers

In the literature, CSF diagnostic biomarkers of iNPH are proposed after comparison with healthy controls and/or AD patients, due to similarities in the cognitive decline, pathological pathways, and overlapping ratings of these conditions. The CSF diagnostic biomarkers proposed for iNPH and their comparison with AD and healthy controls are reported in Table 2.

One of the first papers suggesting a diagnostic role for a CSF BM for iNPH was a case-control study by Li and others. They reported that transforming growth factor (TGF) β1, TGF- β type II receptor (TbR-II), and LRG CSF levels in iNPH patients were significantly higher than in healthy controls, also documenting a significant correlation between TbR-II levels and LRG and TGF-β1 levels. The authors concluded that CSF evaluation of these biomarkers could be useful in iNPH diagnosis because of their hypothetical pathogenetic role in its development (Li et al., 2007) [6]. Kapaki et al. were the first to compare CSF biomarkers in AD patients with those of iNPH patients and healthy controls in 2007. They reported total tau (τ_T_) was significantly increased in iNPH compared with controls but lower than in AD and correlated with Hakim’s triad. Phosphorylated tau (τP−181) levels were normal in iNPH but increased in the AD group. Lastly, Aβ42 decreased in both diseases but mostly in AD. They concluded that Tau protein levels, P-tau in particular, and their ratio can slightly help discriminate between iNPH and AD. (Kapaki et al., 2007) [7] (Ghosh & Lippa., 2014) [8] (Panciani et al., 2025) [9] Schirinzi et al. found lower p-tau levels in iNPH patients than in controls and suggested Aβ42/p-tau ratio as a diagnostic BM, as it was found to be lower in AD than in iNPH. (Schirinzi et al., 2015) [10] CSF Aβ42+ with negative Aβ42/Aβ40, p-tau, and total tau is a typical CSF profile of iNPH. Conversely, positive Aβ42/Aβ40 associated with positive p-tau may lead to suspicion of a coexistent iNPH-AD (Mazzeo et al., 2022) [11]. Patients with progressive supranuclear palsy (PSP) had a similar iNPH CSF profile. (Schirinzi et al., 2015) [10]. Said et al. additionally reported that these BMs can be used to differentiate iNPH even from frontotemporal dementia (FTD) and Lewy Body Dementia (LBDs). (Said et al., 2022) [12] Nakajima’s group also highlighted that LRG levels in iNPH CSF samples are potential diagnostic biomarkers, especially useful in differential diagnosis among iNPH and Parkinson’s disease (PD) with dementia and PSP, which are highly increased (Nakajima et al., 2011) [13] (Miyajima M, 2013) [14]. Vanninen et al. analyzed CSF samples and frontal cortical biopsies, concluding that LRG is a sensitive biomarker for probable iNPH but cannot be used alone to predict a shunt response. Moreover, LRG levels seem not to be affected by the patients’ AD pathology as detected in brain biopsies (Vanninen et al., 2021) [15]. In general, the previously cited CSF biomarkers have differential diagnostic significance in iNPH from its mimics (Jeppsson A, 2019) [16], but they are not automatically diagnostic for iNPH. Pyykko et al. found CSF amyloid precursor protein (APP) α levels to be lower in iNPH patients, suggesting an independent role from the amyloid cascade of sAPP isoforms in iNPH (Pyykko, et al., 2014) [17]. However, a reduction in all APP-derived proteins is also found in small subcortical vessel disease (SSVD) CSF. In contrast, elevations of NFL, myelin basic protein (MBP), glial fibrillary acidic protein (GFAP) and matrix metalloproteinase (MMP)-10 are shared both in SSVD and iNPH compared to healthy controls (Jeppsson et al., 2022) [18]. According to Nishida et al., L-PGDS can be considered a diagnostic biomarker because of its low concentrations in iNPH CSF, and it may also work as a surrogate marker for DESH features. Indeed, L-PGDS levels correlated positively with age and callosal angle but negatively with FAB scores, suggesting a narrow callosal angle, white matter damage detected on imaging, and frontal lobe dysfunction. Moreover, L-PGDS and t-tau levels were correlated and both significantly decreased in DESH patients, while Aβ concentrations did not correlate with clinicoradiological features (Nishida et al., 2014) [19] (Nishida et al., 2014) [20]. CSF NFL level seems to correlate to white matter hyperintensity volume as well (Yang et al., 2023) [21].

More authors reported similar results in their studies (Vanninen et al., 2023) [22] (Klemke et al., 2023) [23] (Jingami et al., 2015) [24] (Chen Z, 2017) [25] suggesting the hypothesis of periventricular amyloid mismetabolism due to glymphatic dysfunction underlying etiology (Jeppsson et al., 2016) [26] (Kim HJ, 2019) [27] (Müller-Schmitz et al., 2020) [28] (Masri et al., 2024) [5] (Nishida et al., 2024) [29]. Even proteomic iNPH profiles showed significant downregulation of synaptic markers and cell–cell adhesion proteins alongside inflammatory marker upregulation, suggesting ependymal dysfunction (Kamalian, 2024) [30] (DeGeus et al., 2025) [31].

The role of proinflammatory cytokines such as several interleukins (IL), interferon-gamma (IFN), monocyte chemoattractant protein-1 (MCP-1), and biomarkers of neuronal damage like NFL and MBP was also investigated. (Pyykko, et al., 2014) [17]. High MCP-1 and CCL4, and low PD-L1 levels may have diagnostic potential in the work-ups of patients with iNPH (Braun, et al., 2023) [32]. Lolarsen found increased IL-6, IL-1β, LRG, IL-18, vascular endothelial growth factor (VEGF), and IFN-γ (Lolansen et al., 2021) [33], but also C–C motif chemokine ligand 28-23-3 (CCL28, CCL23, CCL3), osteoprotegerin (OPG), C-X-C motif chemokine ligand 1 (CXCL1), IL-18, IL-8, Oncostatin-M (OSM), eukaryotic translation initiation factor 4E-binding protein 1 (4E-BP1), C-X-C motif chemokine ligand 6 (CXCL6), and fms-related tyrosine kinase 3 ligand (Flt3L) and decreased CUB domain-containing protein 1 (CDCP1) in iNPH patients compared to control subjects (Lolansen et al., 2021) [34].

To translate these molecular insights into clinical practice, biomarker interpretation in suspected idiopathic normal pressure hydrocephalus (iNPH) must account for the complex physiological interplay between dynamic CSF clearance failure and subcortical structural injury. Disruption of the glymphatic system—facilitated by perivascular channels and astrocytic aquaporin-4 (AQP4) expression—plays a pivotal role in the pathogenesis of both iNPH and Alzheimer’s disease (AD). However, their underlying biomarker profiles reflect distinct pathophysiological mechanisms. While parenchymal Aβ42 sequestration drives cortical amyloidosis in AD, reduced CSF Aβ42 in iNPH primarily stems from impaired interstitial fluid flushing and reduced transependymal bulk flow. Crucially, pure iNPH typically demonstrates a preserved Aβ_42_/Aβ_40_ ratio along with low or normal phosphorylated tau levels, contrasting sharply with the severe tau hyperphosphorylation characteristic of AD. Furthermore, markers of axonal strain such as neurofilament light chain (NfL) and neuroinflammatory mediators capture ongoing subcortical white matter stress and ependymal injury. Evaluating these biomarkers within a combined multi-analyte matrix rather than as isolated measurements allows clinicians to effectively differentiate pure iNPH from comorbid AD pathologies, refine patient selection for surgical interventions, and establish realistic post-shunting outcome expectations.

Torretta et al. additionally evaluated a panel of proteins and sphingolipids such as phosphatidylcholine (PC) 36:2, sphingomyelins (SM) C24:1, sphingosine-1-phosphate (S1P), and glucosylceramide (GlcCer) that can contribute to improving the differential diagnosis of patients with iNPH or AD (Torretta, 2018) [35]. The iNPH profile seems to be characterized by increased very long chains like ceramides (Cer C22:0, Cer C24:0 and Cer C24:1) and acute-phase proteins, immunoglobulins, and complement component fragments. While synaptic signaling and axogenesis, secretory, glycosylation, and proteins involved in lipid metabolism were lower in iNPH (Torretta et al., 2021) [36], the iNPH CSF lipid profile showed a different pattern compared with healthy controls, especially regarding triacylglycerols (TGs) elevation (Toft-Bertelsen et al., 2024) [37]. Porru proposed oxysterols in CSF as diagnostic of iNPH BM, reporting cholesterol, 24-OH, and 27-OH significantly lower in iNPH compared to healthy controls, suggesting the role of the blood–brain barrier integrity in iNPH pathogenesis (Porru et al., 2022) [38]. CSF soluble triggering receptor expressed on myeloid cells 2 (sTREM2), correlating with plasma sTREM2 levels, is not decreased in iNPH subjects as in AD (Ferri et al., 2021) [39]. Lectin-TfAb ELISAs are promising high-throughput methods for diagnosing iNPH due to higher CSF Sambucus sieboldiana agglutinin (SSA)-reactive transferrin (Tf) measuring Tf-2 than in non-iNPH patients (Shirotani et al., 2011) [40]. Also, increased protein tyrosine phosphatase receptor type Q (PTPRQ) CSF values were suggested as diagnostic of iNPH and related to ependymal dysfunction from ventricular expansion (Nagata Y, 2017) [41] (Nakajima M, 2021) [42]. Also, aquaporins AQP1 and AQP4 in CSF were suggested as potential diagnostic biomarkers for iNPH (Hiraldo-González L, 2021) [43]. In addition, the iNPH CSF metabolomic pattern shows altered amino acid metabolism, mitochondrial function, oxidative stress, and neurotransmission (Hofling et al., 2026) [44].

**Table 2:** iNPH CSF diagnostic biomarkers and comparison with AD and healthy controls for differential diagnosis.

Study	Method	CSF Biomarker	AD	iNPH	Healthy Controls	*p*-Value p1 (iNPH vs. Healthy Controls) p2 (iNPH vs. AD)
Li et al., 2007 [6]	ELISA	TGF-β1	n.r.	1439.3 ± 149.2 pg/mL	492.1 ± 34.7 pg/mL	<0.001 (p1)
		TGF-β2	n.r.	137.2 ± 18.6 pg/mL	148.8 ± 18.3 pg/mL	>0.1 (p1)
		TβR-II	n.r.	1992.3 ± 134.1 pg/mL	1065.7 ± 87.8 pg/mL	<0.001 (p1)
		LRG	n.r.	1047.2 ± 63.4 pg/mL	520.4 ± 49.5 pg/mL	<0.001 (p1)
Kapaki et al., 2007 [7]	ELISA	τ_T_	571 (443–984) pg/mL	219 (118–280) pg/mL	166 (117–233) pg/mL	<0.000001 (p1) (p2)
		τ_P−181_	72 (60.4–100) pg/mL	34.8 (20–46.7) pg/mL	45.1 (40–55.8) pg/mL	<0.00001 (p1) <0.001 (p2)
		Aβ42	422 ± 149 pg/mL	400 ± 219 pg/mL	721 ± 228 pg/mL	<0.000001 (p1) <0.00001 (p2)
		τ_T_/Aβ42	1.53 (0.93–2.4) pg/mL	0.55 (0.38–1.24) pg/mL	0.23 (0.16–0.31) pg/mL	<0.00001 (p1) (p2)
		τ_P−181/_τ_T_	0.14 (0.11–0.15) pg/mL	0.16 (0.1–0.19) pg/mL	0.24 (0.21–0.28) pg/mL	<0.001 (p1) <0.01 (p2)
		τ_P−181/_Aβ42	0.19 (0.14–0.26) pg/mL	0.09 (0.07–0.2) pg/mL	0.06 (0.04–0.07) pg/mL	<0.0001 (p1) (p2) <0.001 (p2)
Nakajima et al., 2011 [13]	ELISA	LRG	n.r.	96.8 ± 44.6 ng/mL	n.r.	n.r.
		τ_T_	n.r.	167.4 ± 105.4 pg/mL	n.r.	n.r.
Miyajima et al., 2013 [14]	ELISA	LRG	95.2 ± 64.4 ng/mL	106 ± 46.7 ng/mL	44 ± 25.2 ng/mL	<0.001 (p1)
		τ_T_	416.7 ± 302 pg/mL	149.7 ± 105 pg/mL	155.9 ± 92.5 pg/mL	1.000 (p1)
		τ_P−181_	63.7± 39.3 pg/mL	26.8 ± 13.4 pg/mL	29.2 ± 9.1 pg/mL	0.976 (p1)
		Aβ42	292 ± 244.3 pg/mL	317.9 ± 232.7 pg/mL	324 ± 223 pg/mL	1.000 (p1)
		τ_P−181/_Aβ42	0.75 ± 1.7 pg/mL	0.14± 0.17 pg/mL	0.10 ± 0.06 pg/mL	0.590 (p1)
Pyykko, et al., 2014 [17] *	ELISA	τ_T_	294 (164) ng/L	239 (156) ng/L	n.r.	n.r.
		τ_P−181_	47.1 (13.6) ng/L	35.3 (15.5) ng/L	n.r.	n.r.
		Aβ42	584 (248) ng/L	587 (141) ng/L	n.r.	n.r.
		sAPP-α	325 (238) ng/L	217 (156) ng/L	n.r.	<0.05 (p2)
		sAPP-β	108 (67.9) ng/L	84.3 (63.4) ng/L	n.r.	>0.05 (p2)
		IL-8	461 (592) ng/L	1101 (2440) ng/L	n.r.	n.r.
		TNF-α	2.56 (3.45) ng/L	4.38 (7.80) ng/L	n.r.	n.r.
		NFL	2007 (867) ng/L	2511 (1798) ng/L	n.r.	n.r.
		MCP-1	2618 (2881) ng/L	3398 (2865) ng/L	n.r.	n.r.
		MBP	49.9 (57.7) ng/L	117 (170) ng/L	n.r.	n.r.
Nishida et al., 2014 [19]	ELISA	τ_T_	n.r.	DESH 319.9 ± 31.4 pg/mL	>546.2 ± 54.2 pg/mL	0.003 (p1)
				Non-DESH 546.2 ± 54.2 pg/mL		
		Aβ42	n.r.	DESH 59.2 ± 7.5 pM	90 ± 8 pM	0.84 (p1)
				Non-DESH 57.2 ± 6.0 pM		
		Aβ40	n.r.	DESH 1198 ± 76 pM	1500 ± 50 pM	0.19 (p1)
				Non-DESH 1314 ± 45 pM		
		L-PGDS	n.r.	DESH: 14.4 ± 1.0 μg/mL	15 ± 1.0 μg/mL	0.01 (p1)
				non-DESH: 20.8 ± 2.0 μg/mL		
Lim et al., 2014 [45]	ELISA	Aβ42	409.2 ± 166.1 pg/mL	579.8 ± 182.3 pg/mL	691.8 ± 212.7 pg/mL	0.013 (p2)
		T-tau	259.6 ± 161.5 pg/mL	131.9 ± 77.6 pg/mL	196.9 ± 114.4 pg/mL	0.003 (p2)
		P-tau	51.3 ± 28.3 pg/mL	27.0 ± 9.6 pg/mL	43.0 ± 28.5 pg/mL	0.002 (p2)
Schirinzi et al., 2015 [10]	ELISA	Aβ42	Mean 308.43 pg/mL	Mean 477.5 pg/mL	Mean 862.86 pg/mL	0.01 (p1) (p2)
		T-tau	Mean 662.79 pg/mL	Mean 183.36 pg/mL	Mean 231.14 pg/mL	<0.01 (p1) (p2)
		P-tau	Mean 77.71 pg/mL	Mean 25.36 pg/mL	Mean 40.5 pg/mL	<0.01 (p1) (p2)
		Aβ42/P-tau	Mean 4.26 pg/mL	Mean 18.77 pg/mL	Mean 23.41 pg/mL	<0.01 (p2)
Jingami et al., 2015 [24]	ELISA	LRG	33.0 (28.5–34.2) ng/mL	33.2 (29.1–35.6) ng/mL	n.r.	>0.1 (p2)
		τT	932 (716–1533) pg/mL	297 (220–398) pg/mL	n.r.	<0.0001 (p2)
		τP−181	57.0 (32.1–102) pg/mL	15.7 (11.3–21.5) pg/mL	n.r.	<0.0001 (p2)
		Aβ42/40	0.038 (0.029–0.055)	0.054 (0.044–0.061)	n.r.	<0.01 (p2)
Jeppsson et al., 2016 [26]	ECLIA	Aβ38	n.r.	502 (266–625) ng/L	1114 (819–1445) ng/L	≤0.001 (p1)
		Aβ40	n.r.	3676 (2190–4748) ng/L	7682 (6366–9809) ng/L	≤0.001 (p1)
		Aβ42	n.r.	241 (144–405) ng/L	754 (493–1058) ng/L	≤0.001 (p1)
	ELISA	APL1β25	n.r.	2591 (2296–2951) ng/L	2180 (1898–2386) ng/L	≤0.001 (p1)
		APL1β27	n.r.	1083 (887–1177) ng/L	874 (796–964) ng/L	≤0.001 (p1)
		APL1β28	n.r.	423 (1317–1550) ng/L	1621 (1422–1797) ng/L	≤0.01 (p1)
		NFL	n.r.	1185 (731–2103) ng/L	938 (610–2141) ng/L	≤0.05 (p1)
		YKL-40	n.r.	122 (90–167) ng/mL	137 (104–177) ng/mL	≤0.05 (p1)
Jurjević et al., 2017 [46]	ELISA	sAbPPa	266 (81) ng/mL	157 (61) ng/mL	271 (82) ng/mL	<0.0001 (p2)
		Ab42	433 (173) pg/mL	630 (188) pg/mL	627 (230) pg/mL	0.008 (p2)
		p-Tau	75 (23) pg/mL	27 (9) pg/mL	34 (13) pg/mL	<0.0001 (p2)
		α-syn	0.69 (0.20) ng/mL	0.65 (0.27) ng/mL	n.r.	n.r.
	qRT-PCR	hsa-miR-4274	−9.92 [−10.94∼−7.12]	−8.90 [−10.07∼−7.77]	−6.46 [−7.69∼−5.77]	n.r.
Nagata et al., 2017 [41]	ELISA	PTPRQ	0.35 ng/mL	0.68 ng/mL	0.30 ng/mL	0.0007 (p1) 0.003 (p2)
Torretta et al., 2018 [35]	ELISA	t-tau	774 (208–1420) ± 394 pg/mL	157 (75–284) ± 73.4 pg/mL	103 (49–507) ± 160 pg/mL	n.r.
		p-tau	84 (28–136) ± 31.7 pg/mL	26 (15–42) ±9.2 pg/mL	26 (7–80) ± 24 pg/mL	n.r.
		Aβ	392 (226–563) ± 96 pg/mL	790 (718–1248) ± 267 pg/mL	1082 (453–1515) ± 467 pg/mL	n.r
	MALDI	SM C24:1 (peak mass 766.58 and 835.68 *m*/*z*)	Decreased	Increased	Increased	<0.01 (p1); <0.00001 (p2)
		PC 36:2 (peak mass 794.61, 796.59, 808.61 and 830.61 *m*/*z*)	Decreased	Increased	n.r.	<0.0001 (p2)
	LC-MS	GlcCerC24:0	Increased	Decreased	n.r.	<0.01 (p2)
		S1P	Decreased	Increased	n.r.	<0.05 (p2)
Jeppsson A et al., 2019 [16]	ELISA	τ_T_	980 (333) pg/mL	245 (131) pg/mL	346 (181) pg/mL	<0.001 (p1,p2)
		τ_P−181_	96 (27) pg/mL	32 (12) pg/mL	50 (20) pg/mL	<0.001 (p1,p2)
		Aβ38	2710 (807) pg/mL	1526 (519) pg/mL	2181 (645) pg/mL	<0.001 (p1,p2)
		Aβ40	6541 (1654) pg/mL	3800 (1193) pg/mL	5425 (1510) pg/mL	<0.001 (p1,p2)
		Aβ42	318 (95) pg/mL	364 (138) pg/mL	541 (239) pg/mL	<0.001 (p1)
		sAPP-α	865 (310) pg/mL	446 (178) pg/mL	661 (223) pg/mL	<0.001 (p1,p2)
		sAPP-β	599 (192) pg/mL	321 (121) pg/mL	508 (166) pg/mL	<0.001 (p1,p2)
		MCP-1	436 (162) pg/mL	492 (109) pg/mL	410 (110) pg/mL	<0.05 (p1)
Kim et al., 2019 [27]	ELISA	Aβ42	391.8 ± 120.2 pg/mL	892.8 ± 311.4 pg/mL	1330.0 ± 99.7 pg/mL	<0.001 (p2); 0.089 (p1)
		Aβ40	5462.5 ± 2192.2 pg/mL	5504.0 ± 2867.1 pg/mL	9179.6 ± 2596.9 pg/mL	1.000 (p2); 0.082 (p1)
		Aβ42/Aβ40	0.077 ± 0.020 pg/mL	0.187 ± 0.068 pg/mL	0.155 ± 0.044 pg/mL	0.001 (p2); 0.178 (p1)
		τ_T_	720.7 ± 220.1 pg/mL	238.9 ± 103.7 pg/mL	294.5 ± 95.1 pg/mL	<0.001 (p2); 1.000 (p1)
		τ_P−181_	78.1 ± 30.6 pg/mL	30.4 ± 9.5 pg/mL	36.2 ± 11.1 pg/mL	0.001 (p2); 0.560 (p1)
Vanninen et al., 2021 [15]	ELISA	LRG	n.r.	614.1 ± 418.1 pg/mL	283.0 ± 170.5 pg/mL	>0.001 (p1)
		τ_T_	n.r.	160.3 ± 59.5 pg/mL	252.3 ± 89.2 pg/mL	<0.001 (p1)
		τ_P−181_	n.r.	31.5 ± 11.4 pg/mL	49.4 ± 12.5 pg/mL	<0.001 (p1)
		Aβ42	n.r.	757.3 ± 167.7 pg/mL	933.2 ± 194 pg/mL	<0.001 (p1)
Kawamura et al., 2021 [47]	ELISA	τ_T_	633.4 ± 236.70 pg/mL	106.3 ± 56.38 pg/mL	202.15 ± 166.88 pg/mL	<0.001 (p2)
		τ_P−181_	102.3 ± 35.5 pg/mL	27.0 ± 7.29 pg/mL	25.8 ± 8.7 pg/mL	<0.001 (p2)
		Aβ42	388.8 ± 149.9 pg/mL	589.6 ± 241.2 pg/mL	676.0 ± 193.9 pg/mL	<0.05 (p2)
		τ_P−181/_Aβ42	0.292 ± 0.120 pg/mL	0.055 ± 0.030 pg/mL	0.040 ± 0.014 pg/mL	<0.001 (p2)
		AβO [8,39,43,44,48,49,50,51,52,53,54]	6.01 ± 1.18 pM	6.90 ± 1.45 pM	3.53 ± 1.20 pM	<0.001 (p1)
Nakajima et al., 2021 [42]	ELISA	PTPRQ	365 (225) pg/mL	619 (398) pg/mL	296 (94) pg/mL	0.001 (p1), 0.077 (p2)
		Aβ42	420 (145) pg/mL	500 (236) pg/mL	633 (217) pg/mL	0.533 (p1), 0.725 (p2)
		τ_P−181_	103 (38.5) pg/mL	25.6 (13.0) pg/mL	25.1 (7.5) pg/mL	1.000 (p1), <0.001 (p2)
		τ_T_	595 (276) pg/mL	117 (90.7) pg/mL	170 (86) pg/mL	0.513 (p1), <0.001 (p2)
Hiraldo-González et al., 2021 [43]	ELISA	AQP1	0.07 ± 0.11 ng/mL	0.08 ± 0.07 ng/mL	0.08 ± 0.11 ng/mL	n.r.
		AQP4	0.009 ± 0.01 ng/mL	0.002 ± 0.005 ng/mL	0.08 ± 0.11 ng/mL	n.r.
Ferri et al., 2021 [39]	ELISA	Aβ42	486.5 (136.8) pg/mL	748.8 (312.3) pg/mL	n.r	<0.01 (p2)
		τ_T_	721.9 (524.9) pg/mL	173.3 (101.8) pg/mL	n.r.	<0.01 (p2)
		τ_P−181_	89.6 (42.6) pg/mL	34.2 (13.5) pg/mL	n.r.	<0.01 (p2)
		sTREM2	32.6 (18.1) ng/mL	36.4 (13.2) ng/mL	n.r.	<0.01 (p2)
Lolansen et al., 2021 [34] ^§^	PEA	CCL28	n.r.	1.749 ± 0.222	1.495 ± 0.265	0.0007 (p1)
		CCL23	n.r.	3.260 ± 0.451	2.993 ± 1.013	0.0051 (p1)
		CCL3	n.r.	4.506 ± 0.433	4.262 ± 0.973	0.0056 (p1)
		OPG	n.r.	10.150 ± 0.529	9.765 ± 0.478	0.0207 (p1)
		CXCL1	n.r.	6.646 ± 0.524	6.408 ± 1.091	0.0211 (p1)
		IL-18	n.r.	2.638 ± 0.595	2.409 ± 1.093	0.0227 (p1)
		IL-8	n.r.	8.497 ± 0.506	8.275 ± 0.787	0.0263 (p1)
		OSM	n.r.	0.726 ± 0.373	0.655 ± 0.915	0.0283 (p1)
		4E-BP1	n.r.	2.763 ± 0.653	2.544 ± 1.177	0.0283 (p1)
		CXCL6	n.r.	4.246 ± 0.637	3.929 ± 1.353	0.0375 (p1)
		Flt3L	n.r.	9.100 ± 0.356	8.909 ± 0.515	0.0402 (p1)
		CDCP1	n.r.	4.576 ± 0.245	4.905 ± 0.556	0.0225 (p1)
Said et al., 2022 [12]	ELISA	Aβ42	643.1 (15.3–4181) pg/mL	812.7 (191.4–3255.7) pg/mL	n.r.	<0.05 (p2)
		Aβ40	7998.3 (63–43,911) pg/mL	9889.2 (63–102,721.5) pg/mL	n.r.	<0.05 (p2)
		τ_P−181_	69.2 (8.9–493.2) pg/mL	45.9 (7–680) pg/mL	n.r.	<0.05 (p2)
		τ_T_	306.7 (47.7–5716.9) pg/mL	213.1 (3.1–5142.2) pg/mL	n.r.	<0.05 (p2)
		Aβ42/41	0.07 (0–10.4) pg/mL	0.09 (0.02–15.2) pg/mL	n.r.	<0.05 (p2)
		Aβ42/τ_T_	1.6 (0.02–19.8) pg/mL	3.6 (0.05–495) pg/mL	n.r.	<0.05 (p2)
Porru et al., 2022 [38]	Nephelometry	Albumin	n.r.	243 (72–529) mg/L	205 (115–483) mg/L	0.547 (p1)
	Mass spectrometry	Cholesterol	n.r.	2.3 (1.3–12) (1 missing) g/mL	3.9 (1.9–7.5) g/mL	<0.001 (p1)
		24-OH	n.r.	1.1 (0.5–4.0) ng/mL	1.9 (0.8–5.4) ng/mL	0.006 (p1)
		27-OH	n.r.	0.7 (0.3–1.7) ng/mL	1.1 (0.4–2.5) ng/mL	0.001 (p1)
		7HOCA	n.r.	14 (6.8–28) ng/mL	17 (10–31) ng/mL	0.186 (p1)
Giannini et al., 2022 [51]	RT-QuIC	α-syn	n.r.	60 (20.5)	n.r.	0.56
	CLEIA	τ_T_	n.r.	184 (137–252) pg/mL	n.r.	0.13
		τ_P−181_	n.r.	28 (20–40) pg/mL	n.r.	0.82
		Aβ42	n.r.	573 (424–777) pg/mL	n.r.	<0.001
Jeppsson et al., 2022 [18]	ECLIA	sAPPα	n.r.	384 (303–593) pg/mL	850 (694–1207) pg/mL	<0.001 (p1)
		sAPPβ	n.r.	227 (170–325) pg/mL	516 (446–664) pg/mL	<0.001 (p1)
		Aβ38	n.r.	1333 (823–1928) pg/mL	2855 (2266–3261) pg/mL	<0.001 (p1)
		Aβ40	n.r.	3541 (2206–5648) pg/mL	7009 (5570–7814) pg/mL	<0.001 (p1)
		Aβ42	n.r.	361 (232–496) pg/mL	693 (510–931) pg/mL	<0.001 (p1)
		MMP-10	n.r.	49 (38–67) pg/mL	42 (31–49) pg/mL	<0.05 (p1)
	ELISA	NFL	n.r.	1592 (1012–2519) pg/mL	889 (694–1072) pg/mL	<0.001 (p1)
		GFAP	n.r.	876 (659–1146) pg/mL	559 (381–718) pg/mL	<0.001 (p1)
		MBP	n.r.	1997 (1407–2503) pg/mL	1446 (1228–1632) pg/mL	<0.001 (p1)
Vanninen et al., 2023 [22]	ELISA	τ_T_	408.29 (141.87) pg/mL	144.76 (33.44) pg/mL	185.83 (42.35) pg/mL	<0.05 (p1)
		τ_P−181_	39.92 (17.53) pg/mL	10.27 (3.90) pg/mL	16.84 (5.24) pg/mL	<0.05 (p1)
		Aβ42	602.70 (154.28) pg/mL	964.96 (278.95) pg/mL	1145.60 (237.73) pg/mL	<0.05 (p1)
Klemke et al., 2023 [23]	ELISA	Total CSF protein	Mean 600 mg/L	Mean 450 mg/L	n.r.	<0.05 (p2)
		S100	Mean 2800 pg/mL	Mean 3400 pg/mL	n.r.	<0.05 (p2)
		NSE	Mean 12.5 μg/mL	Mean 12.8 μg/mL	n.r.	n.r.
		τ_T_	Mean 320 pg/mL	Mean 240 pg/mL	n.r.	<0.05 (p2)
		τ_P−181_	Mean 35 pg/mL	Mean 32 pg/mL	n.r.	n.r.
		Aβ42	Mean 450 pg/mL	Mean 800 pg/mL	n.r.	<0.001 (p2)
Yang et al., 2023 [21]	ELISA	τ_T_	n.r.	138.23 ± 6.78 pg/mL	150.66 ± 6.09 pg/mL	0.028 (p1)
		τ_P−181_	n.r.	41.78 ± 5.84 pg/mL	54.77 ± 6.91 pg/mL	0.02 (p1)
		Aβ42	n.r.	188.42 ± 73.62 pg/mL	656.92 ± 56.98 pg/mL	0.033 (p1)
	mECL	NFL	n.r.	1610.32 ± 290.38 pg/mL	764.19 ± 201.55 pg/mL	0.002 (p1)
Nishida et al., 2024 [29]	ELISA	τ_T_	n.r.	251.8 ± 148.8 pg/mL	n.r.	n.r.
		Aβ40	n.r.	56.2 ± 24.3 pg/mL	n.r.	n.r.
		Aβ42	n.r.	1360 ± 429.5 pg/mL	n.r.	n.r.
		L-PGDS	n.r.	16.3 ± 4.3 μg/mL	n.r.	n.r.

* Lumbar CSF values are reported; ^§^ Data are expressed as normalized protein expression (NPX) values (mean ± SD), an arbitrary unit on log2 scale.

#### Co-Pathology CSF Pattern in iNPH

Many studies assessed AD–iNPH coexistence. Pyrgelis et al., in a 2022 narrative review, reported that iNPH patients present lower concentrations of Aβ42 and p-tau than healthy individuals and lower concentrations of t-tau and p-tau than AD patients (Pyrgelis et al., 2022) [55]. In their following studies, they suggested that increased CSF p-tau concentration possibly reflects AD coexistence in iNPH, while higher Aβ42/Aβ40 ratios were found in tap test responders (Pyrgelis & al., 2023) [56]. Moreover, AD coexistence is not uncommon in iNPH patients, and it is associated with worse neuropsychological performances (Pyrgelis, 2024) [57] (Lim et al., 2014) [45] (Müller-Schmitz et al., 2020) (Giannini G., 2022) [51] and exacerbates dysexecutive behaviors. (Niermeyer et al., 2020) [58] Elobeid et al. assessed AD–iNPH coexistence using MMSE scores, AD CSF biomarkers, and frontal cortical biopsies. They reported a significant correlation among all those variables, in particular Aβ42 and hyperphosphorylated tau (HPtau) CSF and cortical biopsies correspondence indicating AD (Elobeid et al., 2015) [49]. An emerging aspect is the underlying α-synuclein pathology that can also coexist with iNPH, estimated in about 30% of iNPH patients, presenting a distinct synSAA+ phenotype with worsening motor symptoms with a PD pattern (Giannini G., 2022) [51] (Weber et al., 2025) [59] (Wluka et al., 2026) [60]. Finally, iNPH patients with a co-pathology should receive a personalized, patient-centered approach.

### 4.2. iNPH Predictive CSF Biomarkers

In 2004, Fersten et al. conducted one of the first studies in the literature referring to CSF biomarkers as outcome predictors in iNPH patients. Although population selection and statistical analysis were unclear, thiobarbituric acid-reactive material (TBAR) and soluble protein sulfhydryl (SH) levels were doubled. In contrast, SH protein levels were lower in the iNPH group than in healthy controls. On this basis, they assumed these CSF biomarkers can be considered positive outcome predictors (Fersten et al., 2004) [50]. Among all the CSF biomarkers tested by Tullberg et al. in 2008, they reported only preoperative higher NFL levels as significant markers of positive surgical outcome and neurological reversibility (Tullberg et al., 2008) [61].

Patel et al. suggested a low CSF p-tau/Aβ1-42 ratio as a predictive favorable outcome biomarker, as well as its role as a marker of comorbid BM of AD (Patel et al., 2012) [62]. Nakajima et al. showed a predictive role of p-tau levels, Aβ1–38/Aβ1–42 ratio, and the Aβ1–42/p-tau ratio in preoperative CSF, while rising CSF L-PGDS and cystatin C were found after shunt treatment anyway (Nakajima et al., 2015) [63]. In 2018, they concluded that preoperative CSF low p-tau levels predicted long-term cognitive improvement after shunting in patients with iNPH. Preoperative high p-tau levels, found in iNPH patients with coexisting AD, correlated with short-term improvement (Nakajima M, 2018) [64]. Craven et al. suggested that, in general, iNPH exhibits low levels of CSF T-tau, which are considered good predictors of outcome (Craven, 2017) [65] (Shir et al., 2026) [66]. Hua et al. concluded that decreased levels of total and phosphorylated tau and a relatively higher ratio of p/Ttau were predictive of good outcomes (Hua R, 2021) [67]. CSF T-tau and P-tau181 appear to be the best predictors (Lukkarinen et al., 2022) [68]. Also, iNPH patients with a higher Aß42/Aß40 ratio demonstrated better cognitive outcomes post-surgery (Spielmann et al., 2025) [69] (Kanemoto et al., 2023) [70]. Lower NfL, pTau181, tTau, and Aβ1–40 were individually predictive of long-term improvement in gait outcomes after shunt surgery (Darrow et al., 2022) [71]. A gradual increase in Aβ 1-42 concentration during three-day external lumbar drainage represents a possible positive prognostic factor (Mandic et al., 2024) [72]. Jingami et al. conducted a dynamic study comparing biomarkers in the first and last drip of CSF samples collected during a preoperative tap test, concluding that t-tau and LRG in the last drip sample are favorable predictors and reflect VCSF dynamic changes (Jingami N, 2019) [73]. Systematic reviews concerning iNPH CSF biomarker predictive value were conducted during the years. Pfanner et al. concluded that Aβ42, tau, p-tau, NFL, and LRG have the greatest amount of evidence for their predictive value. At the same time, TGF-β1, TBR-II, homocysteine, and interleukins show minimal evidence (Pfanner T, 2018) [74]. In particular, CSF levels of P-Tau and total-Tau are significantly increased in shunt-non-responsive iNPH compared to shunt-responsive iNPH (Thavarajasingam, 2022) [75]. Higher levels of NfL and T-tau are associated with a less favorable response to shunt surgery (Braun et al., 2022) [76], contrasting with the earlier finding of Tullberg et al. [61], who reported higher preoperative NFL as a predictor of a favorable surgical outcome. This discrepancy suggests that the direction of the association between preoperative NFL and shunt outcome remains unsettled. However, no single biomarker alone demonstrated a strong predictive value for shunt responsiveness (Cihlo et al., 2025) [77].

Minta et al. (2021) [78] measured iNPH lumbar and ventricular CSF (VCSF) before and after shunt surgery. Even though a general increase in ECM proteins after surgery was found, there was a weak correlation with iNPH. Therefore, shunt surgery led to normalized CSF flow, but ECM proteins are not reliable markers for outcome prediction. Ventricular TIMP-1 was negatively correlated with overall iNPH performance. MMP-9 showed different dynamics compared with other MMPs. Brevican and neurocan peptide concentrations were increased in iNPH patients with cardiovascular disease but not affected by surgery, suggesting no evident dysregulated proteolysis (Minta et al., 2021) [78]. CSF from communicating hydrocephalus shows lower levels of vimentin (VIM), protocadherin alpha subfamily C2 (PCDHAC2), glutathione synthetase (GSS), and prolyl 4-hydroxylase subunit beta (P4HB) than in control subjects (Rostgaard et al., 2023) [79]. A higher preoperative ventricular CSF level of neurogranin (NRGN), a postsynaptic marker, may signal a favorable postoperative outcome (Grønning et al., 2023) [53]. Patel also tested neuronal pentraxin 2 (NPTX2) levels as an outcome predictor. NPTX2 is implicated in glutamatergic signaling and involved in synaptic transmission and plasticity, so it is a proven biomarker of synaptic integrity in cognitive disorders. They reported that even if CSF NPTX2 values correlate with neurodegeneration, they do not reflect cognitive or functional iNPH deficits and are not useful as a predictor of BM (Patel et al., 2024) [80]. Proteomics profiling identified that the pathophysiology of iNPH is characterized by neuroinflammation and neurodegeneration. CSF QPCT and RBP4 were demonstrated to be potential protein biomarkers of shunting prognosis in iNPH (Ying et al., 2026) [81]. The postoperative rise in AQP4 levels and its modest association with clinical improvement, together with lower preoperative levels in shunt responders, indicate that it may help identify shunt-responsive patients (DeRyst et al., 2026) [48]. Tseng et al. tested soluble platelet-derived growth factor receptor-β (sPDGFR-β), known as a BBB leakage marker; its higher levels correlated with iNPH coexisting with CSVD severity and positive surgical outcome (Tseng, 2024) [82]. In iNPH patients compared to controls, there was a higher neutrophil-to-lymphocyte ratio (NLR), directly associated with t-tau and the Evans index, while inversely associated with Aβ42. This proinflammatory state is associated with poor clinical outcomes. Therefore, NLR emerges as a potential prognostic index (Bissacco et al., 2024) [83]. Cystatin-D (CST5) emerged as the only protein significantly associated with postoperative improvement after shunt surgery; it is involved in extracellular matrix remodeling and barrier integrity (Braun et al., 2026) [84]. According to Weiner et al. FABP3, MIF, ANXA4, B3GAT2, ITGB1, YWHAG, OLFM2, TGFBI, and DSG2 are promising predictors of favorable clinical outcomes (Weiner et al., 2023) [85].

CSF outcome predictive biomarkers proposed for iNPH as useful to predict shunt responsiveness are reported in Table 3.

### 4.3. Dual-Function and Other Useful iNPH CSF Biomarkers

Some studies evaluated iNPH CSF BM, proposing them as both diagnostic and predictive of outcome. Kawamura suggested that Aβ oligomers can be both diagnostic and prognostic BM for iNPH because of their high levels that decrease after shunting (Kawamura K, 2021) [47]. Jurjević et al. suggested the role of hsa-miR-4274 as both a diagnostic and predictive CSF BM. In fact, they concluded this miRNA is useful for an accurate iNPH differential diagnosis from AD and especially from Parkinsonian syndromes, as well as a favorable outcome predictor (Jurjević et al., 2017) [46]. Recent reviews concluded the potential role of diagnostic and predictive CSF BMs, especially for total tau, p-tau, and Aβ42 (Nakajima M, 2021) [87] (Yasar & Tullberg, 2025) [88]. Concordance of these BMs between intraventricular and lumbar CSF is reported (Lukkarinen et al., 2023) [86]. However, even if different biomarkers have been identified, combined biomarker panels should be integrated in standardized protocols to have replicable results and an interdisciplinary, physics-based, and patient-centered approach (Kaya & Isik., 2023) [89] (Schmidt & Krauss., 2025) [90].

Other studies have described iNPH CSF biomarkers, not proposing them as diagnostic or predictive of outcome. However, they increase knowledge about the iNPH CSF profile. Methodologically, Konen et al. highlighted the impact of CSF sample fraction choice in these studies, suggesting use of the first 5 mL CSF fraction for analysis to improve diagnostic validity (Konen & Lange, 2022) [91]. The osmolality of lumbar CSF is a reliable reflection of the ventricular CSF osmolality, which is not elevated in iNPH patients (Oernbo et al., 2022) [92]. CSF BM concentrations can also be affected by diseases other than neurodegenerative diseases, such as OSAS (Regalbuto et al., 2024) [93]. The overall proteomic profile differs between the ventricular and the lumbar CSF compartments, and so the biomarkers show an overall protein gradient along the neuroaxis (Rostgaard et al., 2023) [94]. Tarnaris et al. measured various CSF biomarker correlations in iNPH patients at three time points during 72 h of lumbar drainage, documenting a significant lactate, VEGF, GFAP, and tau increase and an NF(h) decrease. Negative correlations were found among GFAP with Aβ42 and total tau, and NF(h) with VEGF (Tarnaris et al., 2009) [95]. Other longitudinal studies were conducted showing time trends of NFL normalization after shunting. At the same time, stable T-tau, P-tau, NRGN, Aβ42 (Lukkarinen, 2021) [96]. Grønning et al. reported postoperative CSF BM concentration changes in: Aβ40, Aβ42, Aβ42/40, sAβPPα and β, NfL, Chitinase-3-like protein 1 (YKL-40), GAP43, MCP-1, p- and T-tau, GFAP, and neurogranin. However, they found p-tau concentration levels did not differ significantly pre- versus post-shunting (Grønning et al., 2025) [52]. CSF analysis did not highlight any differences in beta-amyloid, tau, or phospho-tau in patients with and without freezing of gait (Kihlstedt et al., 2024) [97]. A major dilution effect was not found comparing ventricular volume and lumbar CSF levels of Aβ42, tau, and p-tau biomarkers; subgroup analyses revealed sex-based differences (Lidén & Laurell, 2022) [98]. Saadaldeen et al. reported that high NFL reflecting axonal degeneration was associated with more pronounced iNPH symptoms (Saadaldeen et al., 2025) [99]. Plasma NFL is increased in iNPH patients, reflecting CSF NFL (Jeppsson et al., 2023) [100]. Leinonen in 2011 measured various CSF BM findings, finding that TGFβ1 levels correlated with t-tau and VEGF, and unexpectedly low TNF-α concentrations in iNPH patients (Leinonen et al., 2011) [101]. No significant differences in TNF-α, TGFβ1, VEGF and tau levels were observed between iNPH and subarachnoid hemorrhage (SAH)-induced hydrocephalus, whereas they were both increased compared to obstructive hydrocephalus (Lee et al., 2012) [102]. Recently, Yang et al. reported high concentrations and a positive correlation between CSF neuroinflammation markers YKL-40 and sTREM2, indicating an important role of glial cell interactions in iNPH pathogenesis (Yang et al., 2023) [103]. Brain-derived glycoproteins such as L-PGDS, Man-Tf, and GlcNAc-Tf CSF changes over time were also reported, suggesting them as markers for CSF metabolism (Hoshi K., 2023) [54].

### 4.4. Limits

During evidence acquisition, excluding papers because of full text unavailability could have led to selection bias and therefore it can be considered as a limit. Nonetheless, this choice was made in the interest of offering a completely free and accessible way to approach scientific resources. Due to the huge amount of data collected, statistical or methodological heterogeneity precluded a meta-analysis. Still, SWiM (Synthesis Without Meta-analysis) guidelines were used.

## 5. Conclusions

Collecting and analyzing CSF samples from iNPH patients can be advantageous in differential diagnosis with other similar neurodegenerative disorders, especially AD, and therefore a predictor of neurosurgical outcome. However, even if different biomarkers have been identified, combined biomarker panels should be integrated in standardized protocols to have replicable results and an interdisciplinary, physics-based, and patient-centered approach. Aβ oligomers, tau, and p-tau dosage combination is the most informative CSF analysis in iNPH, yet the least analyzed in literature. Total and phosphorylated tau levels are generally normal in iNPH but increased in AD. Aβ42 decreases in both diseases but less in iNPH than in AD. The ADAβ42/p-tau ratio in iNPH is lower than in healthy controls but higher than in AD, helping us differentiate them. Aβ oligomers can be both diagnostic and prognostic BM for iNPH because of their high levels, which decrease after shunting. Interesting evidence suggests an emerging role of AQP4, LRG, NFL, and L-PDGS. Low AQP4 preoperative levels rise postoperatively. LRG levels seem affected by AD pathology, but LRG’s differential diagnostic role is unclear. Low L-PGDS may also work as a surrogate marker for DESH features. Evidence regarding preoperative NFL as an outcome predictor is not univocal: While Tullberg et al. reported higher preoperative NFL as associated with positive surgical outcome and neurological reversibility (Tullberg et al., 2008) [61], Braun et al. [76] found that higher preoperative NFL levels were instead associated with a less favorable response to shunt surgery, possibly reflecting more extensive axonal injury (Braun et al., 2022) [76]. These divergent findings indicate that the predictive direction of preoperative NFL has not yet been consistently established and warrants further investigation. In synthesis, the evidence suggests a combination of these CSF BM can help differentiate iNPH from its mimics and predict surgical outcomes.

## Figures and Tables

**Table 1:** MeSH search comparison.

MeSH Search	Records			
	PubMed	Scopus	Cochrane	Total
“normal pressure hydrocephalus” and “CSF biomarkers”	121	245	4	370
	118 in English	239 in English	4 in English	361 in English
“idiopathic normal pressure hydrocephalus” and “CSF biomarkers”	91	177	0	268
	89 in English	172 in English	/	261 in English

**Table 3:** CSF biomarkers predictive of favorable outcomes in iNPH vs. healthy controls.

Study	Methods	CSF Biomarkers	Results	
			iNPH	Healthy Controls
Fersten et al., 2004 [50]	n.r.	TBAR	0.58 nmol/mg	0.28 nmol/mg
		SH soluble	41.5 nmol/mg	11.3 nmol/mg
		SH protein	34.3 nmol/mg	57.5 nmol/mg
Tullberg et al., 2008 [61]	Nephelometry	Albumin	544 (171) mg/L	n.r.
	Unprecised	HVA	225 (155) nmol/L	n.r.
		5-HIAA	118 (95) nmol/L	n.r.
		HMPG	36 (16) nmol/L	n.r.
		NPY	106 (36) pmol/L	n.r.
		VIP	8.4 (4.9) pmol/L	n.r.
		GABA	67 (44) nmol/L	n.r.
		Sulphatide	208 (91) nmol/L	n.r.
		GD3	38 (22) nmol/L	n.r.
		NFL	1352 (2989) ng/L	n.r.
		τ_T_	229 (160) ng/L	n.r.
Patel et al., 2012 [62]	Multiplex immunoassay	τ_P−181_/Aβ42 ratio	<0.37	n.r.
Nakajima et al., 2015 [63]	ELISA	Aß1–38/Aß1–42	Median 2.0 pg/mL	n.r.
		Aß1–42/p-tau	Median 23.0 pg/mL	n.r.
		P-tau	Median 20.0 pg/mL	n.r.
		L-PGDS	Median 10.5 μg/mL	n.r.
		Cystatin C	Median 1.58 μg/mL	n.r.
Jurjević, et al., 2017 [46]	qRT-PCR	hsa-miR-4274	Non-shunted: −8.90 [−10.07∼−7.77]	−6.46 [−7.69∼−5.77]
			Shunted: −9.6 [−9.9∼−8.5]	n.r.
Craven et al., 2017 [65]	ELISA	τ_T_	LCSF < 196.5 ng/L (303 ± 245)	n.r.
			VCSF < 525 ng/L (600 ± 429)	
Nakajima et al., 2018 [64]	Multiplex immunoassay	P-tau	<30 pg/mL	n.r.
Jingami et al., 2019 [73]	ELISA	τ_T_	108 (78–198) pg/mL	n.r.
		LRG	74 (53–134) pg/mL	n.r.
Minta et al. 2021 [78]	HPLC-MS based	B156	LCSF mean 10 fmol/µL	n.r.
			VCSF mean 10 fmol/µL	n.r.
		N194	LCSF mean 1 fmol/µL	n.r.
			VCSF mean 1 fmol/µL	n.r.
	FIA or ECLIA	MMP-1,	LCSF mean 10 pg/mL	n.r.
			VCSF mean 8 pg/mL	n.r.
		MMP-2,	LCSF mean 15,000 pg/mL	n.r.
			VCSF mean 90,000 pg/mL	n.r.
		MMP-9,	LCSF mean 20 pg/mL	n.r.
			VCSF mean 25 pg/mL	n.r.
		MMP-10	LCSF mean 25 pg/mL	n.r.
			VCSF mean 20 pg/mL	n.r.
		TIMP-1	LCSF mean 150,000 pg/mL	n.r.
			VCSF mean 100,000 pg/mL	n.r.
Hua et al., 2021 [67]	ELISA	τ_T_	191.21 ± 80.52 pg/mL	n.r.
		τ_P−181_	32.38 ± 12.63 pg/mL	n.r.
		τ_P−181/_τ_T_	17.23 ± 0.59%	n.r.
		Aβ42	584.86 ± 303.55 pg/mL	n.r.
Kawamura et al., 2021 [47] *	ELISA	AβO [8,39,43,44,48,49,50,51,52,53,54]	6.90 ± 1.45 pM	n.r.
Braun et al., 2022 [76]	ELISA	NFL	1175 (850–1970) ng/L	n.r.
		τ_T_	200 (145–282) ng/L	n.r.
		τ_P−181_	29 (23–37) ng/L	n.r.
		Aβ42	536 (386–695) ng/L	n.r.
Darrow et al., 2022 [71]	ELISA	NFL	1298 (933–1940) (IQR)	n.r.
		LRG	280 (182–470) (IQR)	n.r.
		τ_P−181_	20.9(17.6–31.0) (IQR)	n.r.
		τ_T_	211 (132–293) (IQR)	n.r.
		Aβ40	746 (496–1000) (IQR)	n.r.
		Aβ42	5988 (4581–8168) (IQR)	n.r.
Kanemoto et al., 2023 [70]	ELISA	Aß42/Aß40 ratio	0.083 (0.036)	n.r.
		τ_T_	293.6 (165.0) pg/mL	n.r.
Weiner et al., 2023 [85]	TMT proteomics	FABP3	Low, imprecise	n.r
		MIF	Low, imprecise	n.r
		ANXA4	High, imprecise	n.r
		B3GAT2	High, imprecise	n.r
		ITGB1	Low, imprecise	n.r
		YWHAG	Low, imprecise	n.r
		OLFM2	Stable, imprecise	n.r
		TGFBI	Stable, imprecise	n.r.
		DSG2	High, imprecise	n.r
Grønning et al., 2023 [53]	ELISA	NRGN	434 (±279)	n.r.
Pyrgelis et al., 2023 [56]	ELISA	τ_T_	194 (157–272.3)	n.r.
		τ_P−181_	26 (17.7–38)	n.r.
		Aβ42	389 (304–609.5)	n.r.
		Aβ42/Aβ40 ratio	0.16 (0.1–0.2)	n.r.
		τ_P−181_/Aβ42 ratio	0.049 (0.037–0.106)	n.r.
Lukkarinen et al., 2023 [86]	ELISA	τ_T_	V-CSF 325.9 (233.5) ng/L	n.r.
			L-CSF 167.6 (63.9) ng/L	n.r.
		τ_P−181_	V-CSF 14.8 (7.4) ng/L	n.r.
			L-CSF 13.2 (5.9) ng/L	n.r.
		Aβ42	V-CSF 498.2 (245.4) ng/L	n.r.
			L-CSF 914.7 (387.0) ng/L	n.r.
Patel et al., 2024 [80]	ELISA	NPTX2	Non-shunted: 584.16 (±472.41)	n.r
			Shunted: 505.61 (±322.38)	
		τ_P−181_	Non-shunted: 35.91 (±27.25)	n.r
			Shunted: 29.78 (±20.34)	
		Aβ40	Non-shunted: 7761.34 (±3158.35)	n.r
			Shunted: 6994.87 (±2964.27)	
		Aβ42	Non-shunted: 865.69 (±385.77)	n.r
			Shunted: 816.48 (±366.64)	
		Aβ42/Aβ40 ratio	Non-shunted: 0.110 (±0.03)	n.r
			Shunted: 0.119 (±0.03)	
		NFL	Non-shunted: 2156.40 (±1679.10)	n.r
			Shunted: 2062.12 (±1832.94)	
Tseng et al., 2024 [82]	ELISA	τ_T_	M:187.0 (135.6–239.1) pg/ml	n.r.
			F: 180.5 (102.6–317.8) pg/ml	
		τ_P−181_	M: 31.3 (24.5–40.3) pg/mL	n.r.
			F: 26.6 (19.4–32.86) pg/mL	
		Aβ42	M: 403.4 (280.6–595.6) pg/mL	n.r.
			F: 384.0 (225.0–736.7) pg/mL	
		sPDGFR-βa	M: 444.3 (403.4–554.7) pg/mL	n.r.
			F: 556.0 (474.3–667.6) pg/mL	
		NfL	M: 287.9 (235.3–341.7) pg/mL	n.r.
			F: 275.5 (227.9–327.3) pg/mL	
		Trem2	M: 15,376.5 (10,137.5–19,383) pg/mL	n.r.
			F: 16,309.0 (9258–22,400.0) pg/mL	
Bissacco et al., 2024 [83]	Blood cell count	NLR	2.43 ± 1.04	1.61 ± 0.47
	ELISA	Aβ42	590.34 ± 309.90 pg/mL	962.30 ± 539.63 pg/mL
		τ_T_	219.30 ± 100.24 pg/mL	249.86 ± 156.17 pg/mL
		τ_P−181_	35.58 ± 21.20 pg/mL	37.12 ± 15.16 pg/mL
Spielmann et al., 2025 [69]	ELISA	Aβ42/Aβ40	10.3 ± 2.1 pg/mL	n.r.
Cihlo et al., 2025 [77]	ELISA	NSE	1.28 (1.07–1.64) ng/mL	n.r.
		NFL	892.60 (488.67–1473.24) pg/mL	n.r.
		Nfh	1189.93 (575.93–2917.06) pg/mL	n.r.
		τ_T_	411.75 (230.72–492.88) pg/mL	n.r.
		S100	212.46 (142.42–449.14) pg/mL	n.r.
		Aβ42	51.13 (30.44–60.48) pg/mL	n.r.
DeRyst et al., 2026 [48]	bead-based assay	AQP4	536 (386–695) ng/L preop.; 1089 (971–1277) ng/L postop.	n.r.
	ELISA	τ_T_	198 (158–267) ng/L	n.r.
		τ_P−181_	30 (24–38) ng/L	n.r.
		Aβ42	421 (330–534) ng/L	n.r.
		GFAP	490 (350–690) ng/L	n.r.

* COHORT-1 data reported.

## Data Availability

No new data were created or analyzed in this study. Data sharing is not applicable to this article.

## Supplementary Materials

The following supporting information can be downloaded at: https://www.mdpi.com/article/10.3390/ijms27208986/s1.
