# Supplementary material for: Diagnostic and Predictive CSF Biomarkers in Idiopathic Normal Pressure Hydrocephalus (iNPH): A Systematic Review
Source: Int J Mol Sci. 2026 Oct 10;27(20):8986. doi: 10.3390/ijms27208986 (PMC13704644; doi:10.3390/ijms27208986)
Supplement: Supplementary file 1 [file ijms-27-08986-s001.zip › ijms-4487155-supplementary v2.pdf]

Supplementary file

Table S1. Summary of iNPH CSF biomakers analyzed in literature

CSF biomarkers	***Study***
TBAR	Fersten et al., 2004
SH	Fersten et al., 2004
TNF-α	Leinonen et al.,2011; Lee et al., 2012; Pyykko, et al., 2014; Kaya & Isik., 2023;
TGF-β1	Li et al., 2006; Leinonen et al.,2011; Lee et al., 2012; Kaya & Isik., 2023;
TGF-β2	Li et al., 2006
TGF-β3	Li et al., 2006
TβR-II	Li et al., 2006; Kaya & Isik., 2023;
IL (1b, 2, 4, 5, 6, 8, 10, 12p70, 13, 18)	Pyykko, et al., 2014; Kaya & Isik., 2023; Lolansen, et al., 2021
IFN	Pyykko, et al., 2014; Lolansen, et al., 2021;
MCP-1	Pyykko, et al., 2014; Jeppsson A, 2019; Lukkarinen, et al., 2022; Braun, et al., 2023; Kaya & Isik., 2023; Grønning, et al.(2023, 2025); Braun, et al., 2026
PD-L1	Braun, et al., 2023;
LRG	Li et al., 2006; Nakajima et al.,2011; Miyajima et al.,2013; Jingamia et al,(2015, 2019); Vanninen et al., 2021; Lolansen, et al., 2021; Darrow, et al., 2022; Kaya & Isik., 2023;
Lactate	Tarnaris et al., 2009
8-isoprostane	Tarnaris et al., 2009
GFAP	Tarnaris et al., 2009; Jeppsson et al., 2022; Kaya & Isik., 2023; Grønning, et al., (2023;2025); DeRyst et al., 2026
GAP43	Grønning, et al.(2023, 2025)
NF (h)	Tarnaris et al., 2009, Cihlo, et al., 2025
NFL	Tullberg et al.,2008; Leinonen et al., 2011; Pyykko, et al., 2014; Jeppsson et al., (2016, 2022, 2023); Lukkarinen et al.,2021; Giannini G., 2022; Darrow, et al., 2022; Braun et al.,2022; Kaya & Isik., 2023; Rostgaard, et al. 2023; Grønning, et al.(2023, 2025); Yang, et al., 2023Patel et al.,2024; Tseng et al.,2024; Kamalian, 2024; ; Cihlo, et al., 2025
MBP	Pyykko, et al., 2014; Jeppsson et al., 2022; Kaya & Isik., 2023;
VEGF	Tarnaris et al., 2009; Leinonen et al., 2011; Lee et al., 2012; Lolansen, et al., 2021;
Brevican peptide	Minta et al. 2021
Neurocan peptide	Minta et al. 2021
MMP (-1,2,3,9,10)	Minta et al. 2021; Jeppsson et al., 2022; Kaya & Isik., 2023;
TIMP-1	Minta et al. 2021; Jeppsson et al., 2022; Kaya & Isik., 2023;
τ_T	Kapaki et al.,2007; Tullberg et al.,2008; Tarnaris et al., 2009; Leinonen et al., 2011; Lee et al., 2012; Miyajima et al.,2013; Pyykko, et al., 2014; Lim et al., 2014; Nishida et al. (2014, 2024); Jingami et al,(2015, 2019); Schirinzi et al. 2015; Craven et al.,2017; Nakajima et al. (2018,2021); Torretta et al.(2018, 2021); Jeppsson A, 2019; Müller-Schmitz, et al., 2020; Vanninen et al., 2021; Kawamura et al., 2021; Lukkarinen et al.,2021; Hua et al., 2021; Ferri, et al., 2021; Braun et al.,2022; Said et al.,2022; Pyrgelis et al.,2022; Giannini G., 2022; Darrow, et al., 2022; Lidén & Laurell, 2022; Lukkarinen, et al., 2022; Pyrgelis et al.,2023; Yang et al. 2023; Grønning, et al.(2023, 2025); Vanninen et al.,2023; Klemke et al.,2023; Lukkarinen, et al., 2023; Kaya & Isik., 2023; Rostgaard, et al. 2023; Kanemoto et al.,2023; Tseng et al.,2024; Bissacco, et al., 2024; Pyrgelis et al. 2024; Kihlstedt, et al., 2024; Regalbuto et al., 2024; Weber et al., 2025; Cihlo, et al., 2025; Spielmann, et al., 2025; De Geus, et al., 2025; Hofling, et al., 2026; DeRyst et al., 2026; Shir, et al., 2026
τ_{P-181}	Kapaki et al.,2007; Leinonen et al., 2011; Patel et al.,2012; Miyajima et al.,2013; Pyykko, et al., 2014; Lim et al., 2014; Jingami et al,2015; Nakajima et al.,(2015,2018,2021); Schirinzi et al. 2015; Jurjević, et al., 2017; Torretta et al.(2018,2021); Müller-Schmitz, et al., 2020; Vanninen et al., 2021; Kawamura et al., 2021; Lukkarinen et al.,2021; Hua et al., 2021; Ferri, et al., 2021; Braun et al.,2022; Said et al.,2022; Pyrgelis et al.,2022; Giannini G., 2022; Darrow, et al., 2022; Lukkarinen, et al., 2022; Lidén & Laurell, 2022; Pyrgelis et al.,2023; Grønning, et al.(2023, 2025); Yang et al. 2023; Vanninen et al.,2023; Klemke et al.,2023; Rostgaard, et al. 2023; Lukkarinen, et al., 2023; Kaya & Isik., 2023; Patel et al.,2024; Bissacco, et al., 2024; Tseng et al.,2024; Pyrgelis et al. 2024; Kihlstedt, et al., 2024; Kamalian, 2024; Regalbuto et al., 2024; Weber et al., 2025; Spielmann, et al., 2025; De Geus, et al., 2025; Hofling, et al., 2026; DeRyst et al., 2026
HPtau	Elobeid et al.,2015;

Aβ38	Pyykko, et al., 2014; Nakajima et al.,2015, Jeppsson et al., (2016, 2019, 2022); Lukkarinen, et al., 2022; Kaya & Isik., 2023; Grønning, et al., 2023
Aβ40	Pyykko, et al., 2014; Nishida et al. (2014, 2024); Jingamia et al,(2015, 2019); Jeppsson et al.(2016, 2019, 2022); Said et al.,2022; Giannini G., 2022; Darrow, et al., 2022; Lukkarinen, et al., 2022; Kanemoto et al.,2023; Pyrgelis et al.,2023; Grønning, et al.(2023, 2025); Kaya & Isik., 2023; Rostgaard, et al. 2023; Patel et al.,2024; Pyrgelis et al. 2024; Kamalian, 2024; Weber et al., 2025; Spielmann, et al., 2025;; De Geus, et al., 2025
Aβ42	Kapaki et al.,2007; Tarnaris et al., 2009; Patel et al.,2012; Pyykko, et al., 2014; Nishida et al. (2014, 2024); Jingamia et al,(2015, 2019); Jeppsson et al.(2016, 2019,2022); Craven et al.,2017; Jurjević, et al., 2017; Torretta et al.(2018,2021); Leinonen et al., 2011; Miyajima et al.,2013; Lim et al., 2014; Nakajima et al.(2015, 2018,2021); Elobeid et al.,2015; Schirinzi et al. 2015; Vanninen et al., 2021; Kawamura et al., 2021; Lukkarinen et al.,2021; Hua et al., 2021; Ferri, et al., 2021; Braun et al.,2022; Said et al.,2022; Lukkarinen, et al., 2022; Pyrgelis et al.,2022; Giannini G., 2022; Darrow, et al., 2022; Lidén & Laurell, 2022; Kanemoto et al.,2023; Grønning, et al.(2023, 2025); Pyrgelis et al.,2023; Yang et al., 2023; Vanninen et al.,2023; Klemke et al.,2023; Lukkarinen, et al., 2023; Kaya & Isik., 2023; Rostgaard, et al. 2023; Patel et al.,2024; Bissacco, et al., 2024; Tseng et al.,2024; Pyrgelis et al. 2024; Kihlstedt, et al., 2024; Kamalian, 2024; Mandic, et al., 2024; Regalbuto et al., 2024; Weber et al., 2025; Spielmann, et al., 2025; De Geus, et al., 2025; Cihlo, et al., 2025; Hofling, et al., 2026; DeRyst et al., 2026
AβO [10-20]	Kawamura et al., 2021;
sAPP (α, β)	Pyykko, et al., 2014; Jurjević, et al., 2017; Jeppsson A, (2019, 2022); Torretta et al., 2021; Lukkarinen, et al., 2022; Kaya & Isik., 2023; Grønning, et al.(2023, 2025);
APL1β25	Jeppsson et al., 2016
APL1β27	Jeppsson et al., 2016
APL1β28	Jeppsson et al., 2016
α-syn	Jurjević, et al., 2017; Giannini G., 2022; Weber et al., 2025; Wluka, et al., 2026
L-PGDS	Nishida et al.(2014, 2014; 2024); Nakajima et al.,2015; Hoshi K et al., 2023
Cystatine C	Nakajima et al.,2015
YKL-40	Jeppsson et al., 2016; Yang et al. 2023; Kaya & Isik., 2023; Grønning, et al.(2023; 2025)
sTREM2	Ferri, et al., 2021; Yang et al. 2023; Kaya & Isik., 2023; Tseng et al.,2024
FABP3	Weiner et al.,2023
MIF	Weiner et al.,2023
ANXA4	Weiner et al.,2023
B3GAT2	Weiner et al.,2023
ITGB1	Weiner et al.,2023
YWHAG	Weiner et al.,2023
OLFM2	Weiner et al.,2023
TGFBI	Weiner et al.,2023
DSG2	Weiner et al.,2023
S100	Müller-Schmitz, et al., 2020; Klemke et al.,2023; Cihlo, et al., 2025
NSE	Müller-Schmitz, et al., 2020; Klemke et al.,2023; Cihlo, et al., 2025
NPTX (1,2,3)	Patel et al.,2024; De Geus, et al., 2025
NPTXR	De Geus, et al., 2025
sPDGFR-βa	Tseng et al.,2024
CDH5	De Geus, et al., 2025
LTBP2	De Geus, et al., 2025
COL1A1	De Geus, et al., 2025
Fibrinogen markers	De Geus, et al., 2025
Follistatin like 1	De Geus, et al., 2025
complement protein 5	De Geus, et al., 2025
ganglioside GM2 activator	De Geus, et al., 2025
Albumin	Tullberg et al.,2008
HVA	Tullberg et al.,2008
5-HIAA	Tullberg et al.,2008
HMPG	Tullberg et al.,2008
NPY	Tullberg et al.,2008
VIP	Tullberg et al.,2008
GABA	Tullberg et al.,2008
Sulphatide	Tullberg et al.,2008
GD3	Tullberg et al.,2008
hsa-miR-4274	Jurjević, et al., 2017; Kaya & Isik., 2023;
PTPRQ	Nagata et al.,2017; Nakajima et al., 2021; Kaya & Isik., 2023;
SM (C24:1; 32:3, 36:1; 36:2)	Torretta et al., (2018, 2021); Toft-Bertelsen, et al., 2024
PC (30:0, 32:0,16:0/16:0, 33:0, 34:1, 34:4, 36:1, 36:1.1, 36:2, 36:6, 38:1, 38:4, 38:6, 39:6.1, 40:4, 40:6, 40:7)	Torretta et al., 2018; Toft-Bertelsen, et al., 2024

PE (38:4,38:6, 40:6)	Toft-Bertelsen, et al., 2024
plasmenyl-PEs (40:6; 43:6)	Toft-Bertelsen, et al., 2024
docosahexaenoyl PAF C-16	Toft-Bertelsen, et al., 2024
PAs (43:4; 45:4)	Toft-Bertelsen, et al., 2024
plasmenyl-PC 40:6	Toft-Bertelsen, et al., 2024
LysoPC 17:0	Toft-Bertelsen, et al., 2024
PS (39:0;39:7)	Toft-Bertelsen, et al., 2024
S1P	Torretta et al., 2018
GlcCer C24:0	Torretta et al. (2018, 2021)
Cer (C22; 20:5)	Torretta et al., 2021
TG (48:2, 14:0/16:1/18:1, 48:3; 50:4, 16:1/ 16:1/18:2; 51:2; 51:4; 52:4, 16:0/18:2/ 18:2; 53:4; 56:6, 16:0/18:1/22:5; 58:6; 58:8; 60:9; 62:11)	Toft-Bertelsen, et al., 2024
NRGN	Lukkarinen et al.,2021; Grønning, et al., (2023;2025)
BACE1	Torretta et al., 2021
VGF	Torretta et al., 2021
SEZ6L, SEZ6L2	Torretta et al., 2021
CHGA	Torretta et al., 2021
POMGNT1	Torretta et al., 2021
DAG1	Torretta et al., 2021
APOH	Torretta et al., 2021
LCAT	Torretta et al., 2021
AQP (1, 4)	Hiraldo-González L, 2021; Kaya & Isik., 2023; DeRyst et al., 2026
VIM	Rostgaard, et al., 2023
PCDHAC2	Rostgaard, et al., 2023
P4HB	Rostgaard, et al., 2023
GSS	Rostgaard, et al., 2023
GBP2	Kamalian, 2024
SPRR1B	Kamalian, 2024
CRYM	Kamalian, 2024
PODXL2	Kamalian, 2024
TAP1	Kamalian, 2024
RTN4R	Kamalian, 2024
RNF5	Kamalian, 2024
JMJD1C	Kamalian, 2024
B4GAT1	Kamalian, 2024
GAD1	Kamalian, 2024
CD99L2	Kamalian, 2024
ZNRF4	Kamalian, 2024
L1CAM	Kamalian, 2024
GlcNAc-Tf	Hoshi K et al., 2023
Man-Tf	Hoshi K et al., 2023
Tf (1,2)	Shirotani, et al., 2011
CCL(28,23,3,4,11)	Lolansen, et al., 2021; Braun, et al., 2023;
OPG	Lolansen, et al., 2021
CXCL (1,6,9)	Lolansen, et al., 2021; Braun, et al., 2026
OSM	Lolansen, et al., 2021
4E-BP1	Lolansen, et al., 2021
Flt3L	Lolansen, et al., 2021
CDCP1	Lolansen, et al., 2021
CST5	Braun, et al., 2026
