# Supplementary material for: Diagnostic and Predictive CSF Biomarkers in Idiopathic Normal Pressure Hydrocephalus (iNPH): A Systematic Review
Source: Int J Mol Sci. 2026 Oct 10;27(20):8986. doi: 10.3390/ijms27208986 (PMC13704644; doi:10.3390/ijms27208986)
Supplement: Supplementary file 1 [file ijms-27-08986-s001.zip › PRISMA_2020_checklist.pdf]

PRISMA 2020 Checklist

Manuscript: *“Diagnostic and Predictive CSF Biomarkers in Idiopathic Normal Pressure Hydrocephalus (iNPH): A Systematic Review”, Procopio et al.*

Based on: Page MJ, McKenzie JE, Bossuyt PM, Boutron I, Hoffmann TC, Mulrow CD, et al. The PRISMA 2020 statement: an updated guideline for reporting systematic reviews. BMJ 2021;372:n71.

Note: Page numbers refer to the current revised manuscript file (with tracked changes displayed) and may shift slightly after final typesetting. Cells highlighted in yellow indicate items the authors should confirm, as the corresponding information could not be located in the main manuscript text.

**Section and Topic**	**Item #**	**Checklist item**	**Location where item is reported**
TITLE	1	Identify the report as a systematic review.	Title page (title: “Diagnostic and Predictive CSF Biomarkers in Idiopathic Normal Pressure Hydrocephalus (iNPH): A Systematic Review”), p. 1
ABSTRACT	2	See the PRISMA 2020 for Abstracts checklist.	Abstract, p. 1
INTRODUCTION	3	Describe the rationale for the review in the context of existing knowledge.	Introduction, §1, p. 2 (paragraph on diagnostic challenge and rationale for identifying CSF biomarkers)
	4	Provide an explicit statement of the objective(s) or question(s) the review addresses.	Introduction, §1, p. 2, final sentence (“Our systematic review aims to analyze current literature to identify possible diagnostic as well as outcome predictive CSF BM for iNPH”)
METHODS	5	Specify the inclusion and exclusion criteria for the review and how studies were grouped for the syntheses.	§2.1 Evidence Acquisition, p. 3–4 (English-language studies; MeSH terms; grouping by diagnostic/predictive/both/neither outcome)
	6	Specify all databases, registers, websites, organisations, reference lists and other sources searched or consulted, and the date each was last searched.	§2.1 Evidence Acquisition, p. 3 (PubMed, Scopus, Cochrane Library; first consulted April 2025, updated May 2026)
	7	Present the full search strategies for all databases, registers and websites, including filters and limits.	§2.1 Evidence Acquisition, p. 3 (MeSH terms “idiopathic normal pressure hydrocephalus” and “CSF biomarkers”; English-language filter); full combined search comparison in Table 1, p. 7
	8	Specify the methods used to decide whether a study met the inclusion criteria, including number of reviewers and whether they worked independently.	§2.1 Evidence Acquisition, p. 3–4 (screened by two authors, E.P. and A.D.T.; eligibility defined on title/abstract, full-text review when available)
	9	Specify the methods used to collect data from reports, including number of reviewers, independence, and any automation tools.	§2.1 Evidence Acquisition, p. 4 (data extracted by one author, E.P., independently reviewed by four authors, A.D.T., C.A.S., G.G., A.A.; final revision by A.L. and D.L.T.)

Section and Topic	Item #	Checklist item	Location where item is reported
	10a	List and define all outcomes for which data were sought.	§2.1 Evidence Acquisition, p. 4 (study outcomes: iNPH CSF BM qualitative/quantitative measures; diagnostic role vs. AD and other conditions; predictor of shunt outcome)
	10b	List and define all other variables for which data were sought.	§2.1 Evidence Acquisition, p. 4 (type of study, aim, population variables, outcomes, methodology)
	11	Specify the methods used to assess risk of bias, including tools used and number/independence of reviewers.	§2.2 Risk of Bias Assessment, p. 4–5 (QUADAS-2, QUIPS, ROBINS-I tools; synthesized with RobVis, version 0.3.0)
	12	Specify for each outcome the effect measure(s) used in the synthesis or presentation of results.	§2.1 Evidence Acquisition, p. 4 (p-value used as the standardized comparative metric, due to heterogeneity of reported parameters)
	13a	Describe the processes used to decide which studies were eligible for each synthesis.	§2.1 Evidence Acquisition, p. 4 (studies grouped by outcome category: diagnostic, predictive, both, neither)
	13b	Describe any methods required to prepare the data for presentation or synthesis.	§2.1 Evidence Acquisition, p. 4 (data summarized and tabulated per outcome group; primary-study data reported in tables, reviews cited narratively to avoid double-counting)
	13c	Describe methods used to tabulate or visually display results of individual studies and syntheses.	Results, Tables 1, 3 and 4 and Figures 1–5, p. 6–9 and throughout §3
	13d	Describe methods used to synthesize results and provide a rationale; if meta-analysis, describe model(s) and software.	§2.1 Evidence Acquisition, p. 3–4 (SWiM – Synthesis Without Meta-analysis – guidelines used in place of meta-analysis, given statistical/methodological heterogeneity, as also noted in Discussion, p. 28)
	13e	Describe any methods used to explore possible causes of heterogeneity among study results.	§2.1 Evidence Acquisition, p. 4 (heterogeneity investigated by informal methods, considering methodological characteristics, subpopulations and contextual factors)
	13f	Describe any sensitivity analyses conducted.	Not performed – not applicable (narrative synthesis without meta-analysis; please confirm)
	14	Describe any methods used to assess risk of bias due to missing results (reporting bias) in a synthesis.	Not explicitly reported – please confirm with authors whether a reporting-bias assessment was performed
	15	Describe any methods used to assess certainty (confidence) in the body of evidence for an outcome.	§2.1 Evidence Acquisition, p. 4 (“The certainty of the evidence was assessed considering the massive number of studies and participants”); combined with risk-of-bias assessment, §2.2

Section and Topic	Item #	Checklist item	Location where item is reported
RESULTS	16a	Describe the results of the search and selection process, ideally using a flow diagram.	§3.1 Evidence Synthesis Results, p. 6–7, and Figure 1 (PRISMA flow diagram), p. 6
	16b	Cite studies that might appear eligible but were excluded, and explain why.	§3.1 Evidence Synthesis Results, p. 6–7 (records excluded for non-English language, duplicate records, or full-text unavailability)
	17	Cite each included study and present its characteristics.	Table 1 (MeSH search comparison), p. 7; Tables 3 and 4 and accompanying narrative, §3, p. 8–27
	18	Present assessments of risk of bias for each included study.	§3.2 Risk of Bias Assessment Results, p. 9, and Figures 3–5 (QUADAS-2, QUIPS and ROBINS-I summary plots)
	19	For all outcomes, present summary statistics and effect estimates for each study.	Tables 3 and 4 (diagnostic and predictive CSF biomarker results with p-values), p. 8–27
	20a	Briefly summarize the characteristics and risk of bias among studies contributing to the synthesis.	§3.2 Risk of Bias Assessment Results, p. 9
	20b	Present results of all statistical syntheses conducted.	§3 Results narrative (§3.1–3.2) and Tables 2–4, p. 6–27; Discussion, §4, p. 10–28
	20c	Present results of all investigations of possible causes of heterogeneity among study results.	§3.2 Risk of Bias Assessment Results, p. 9 (e.g., patient-selection and confounding-bias domains linked to iNPH heterogeneity)
	20d	Present results of any sensitivity analyses conducted.	Not applicable – no sensitivity analyses performed (see item 13f)
	21	Present assessments of risk of bias due to missing results for each synthesis assessed.	Not explicitly reported – please confirm with authors
	22	Present assessments of certainty in the body of evidence for each outcome.	§3.2 Risk of Bias Assessment Results, p. 9, and Discussion/Conclusions, p. 10–28 (overall low risk of bias supporting the reported associations)
DISCUSSION	23a	Provide a general interpretation of the results in the context of other evidence.	§4 Discussion, p. 10–28
	23b	Discuss any limitations of the evidence included in the review.	§4 Discussion, p. 28 (heterogeneity of parameters; conflicting findings, e.g., preoperative NFL direction of association)
	23c	Discuss any limitations of the review processes used.	§4 Discussion, p. 28 (English-language restriction and full-text availability as sources of possible selection bias;

Section and Topic	Item #	Checklist item	Location where item is reported
			statistical/methodological heterogeneity precluding meta-analysis)
	23d	Discuss implications of the results for practice, policy, and/or future research.	§5 Conclusions, p. 28 (proposed combined biomarker panel and call for standardized, replicable protocols)
OTHER INFORMATION	24a	Provide registration information, including register name and number, or state that the review was not registered.	Not reported in the manuscript – please confirm registration status (e.g., PROSPERO) or state that the review was not registered
	24b	Indicate where the review protocol can be accessed, or state that a protocol was not prepared.	Not reported in the manuscript – please confirm or state that no protocol was prepared
	24c	Describe and explain any amendments to information provided at registration or in the protocol.	Not applicable (no registration/protocol reported)
	25	Describe sources of financial or non-financial support, and the role of funders/sponsors.	Funding statement, p. 29 ("This research received no external funding")
	26	Declare any competing interests of review authors.	Conflicts of Interest statement, p. 29 ("The authors declare no conflict of interest")
	27	Report which of the following are publicly available: data collection forms; extracted data; analytic code; other materials.	Data Availability Statement, p. 29 ("No new data were created or analyzed in this study"); CSF biomarker list analyzed is provided in Table S2, Supplementary Materials
